# Serum immunoglobulins and biomarkers of dementia: a population-based study

**DOI:** 10.1186/s13195-023-01333-3

**Published:** 2023-11-07

**Authors:** Amber Yaqub, Samer R. Khan, Meike W. Vernooij, P. Martin van Hagen, Robin P. Peeters, M. Arfan Ikram, Layal Chaker, Virgil A. S. H. Dalm

**Affiliations:** 1 Department of Epidemiology, Erasmus University Medical Center, Rotterdam, the Netherlands; 2 Department of Internal Medicine, Division of Allergy & Clinical Immunology, Erasmus University Medical Center, Rotterdam, The Netherlands; 3 Department of Radiology and Nuclear Medicine, Erasmus University Medical Center, Rotterdam, The Netherlands; 4 Department of Immunology, Erasmus University Medical Center, Rotterdam, The Netherlands; 5 Department of Internal Medicine, Division of Endocrinology, Erasmus University Medical Center, Rotterdam, the Netherlands

**Keywords:** Immunoglobulins, Dementia, Biomarkers, Neuroimaging, Population-based

## Abstract

**Background:**

Inflammation plays a key role in the development of dementia, but its link to early biomarkers, particularly those in plasma or neuroimaging, remains elusive. This study aimed to investigate the association between serum immunoglobulins and biomarkers of dementia.

**Methods:**

Between 1997 and 2009, serum immunoglobulins (IgA, IgG and IgM) were measured in dementia-free participants of the population-based Rotterdam Study. A random subset of participants had assessment of biomarkers in plasma (total tau (t-tau), neurofilament light chain (NfL), amyloid-β40 (Aβ-40), amyloid-β42 (Aβ-42), while another subset of participants underwent neuroimaging to quantify brain volume, white matter structural integrity and markers of cerebral small vessel disease. Linear regression models were constructed to determine cross-sectional associations between IgA, IgG, IgM and biomarkers of dementia, with adjustment for potential confounders. Multiple testing correction was applied using the false discovery rate. As a sensitivity analysis, we re-ran the models for participants within the reference range of immunoglobulins, excluding those using immunomodulating drugs, and conducted a stratified analysis by *APOE*-ε4 carriership and sex.

**Results:**

Of 8,768 participants with serum immunoglobulins, 3,455 participants (65.8 years [interquartile range (IQR): 61.5–72.0], 57.2% female) had plasma biomarkers available and 3,139 participants (57.4 years [IQR: 52.7–60.7], 54.4% female) had neuroimaging data. Overall, no associations between serum immunoglobulins and biomarkers of dementia remained significant after correction for multiple testing. However, several suggestive associations were noted: higher serum IgA levels concurred with lower plasma levels of Aβ-42 (standardized adjusted mean difference: -0.015 [95% confidence interval (CI): -0.029−-0.002], *p* = 2.8 × 10^–2^), and a lower total brain volume, mainly driven by less gray matter (-0.027 [-0.046−-0.008], *p* = 6.0 × 10^–3^) and more white matter hyperintensities (0.047 [0.016 – 0.077], *p* = 3.0 × 10^–3^). In sensitivity analyses, higher IgM was linked to lower t-tau, Aβ-40, and Aβ-42, but also a loss of white matter microstructural integrity. Stratified analyses indicate that these associations potentially differ between carriers and non-carriers of the *APOE*-ε4 allele and men and women.

**Conclusions:**

While associations between serum immunoglobulins and early markers of dementia could not be established in this population-based sample, it may be valuable to consider factors such as *APOE*-ε4 allele carriership and sex in future investigations.

**Supplementary Information:**

The online version contains supplementary material available at 10.1186/s13195-023-01333-3.

## Introduction

Dementia is a multifactorial neurodegenerative syndrome marked by a progressive decline in cognitive ability and loss of independent functioning, with Alzheimer’s disease as the most common form [1]. At a cellular level, pathologic features of Alzheimer’s disease include loss of synaptic function, extracellular deposits of amyloid-β (Aβ plaques) and intracellular aggregates of hyperphosphorylated tau (tangles) [2], which can be present years prior to clinical manifestation of disease. Interestingly, a growing body of literature suggests that dysregulation of the immune system, triggered by inflammatory processes on a peripheral or central level, may contribute to the pathogenesis of dementia [3]. In the context of neuroinflammation, most scientific interest has been garnered towards the innate immune system, a non-specific first line response to a variety of stimuli [4]. For instance, pattern recognition receptors on astrocytes and microglia have been shown to activate an inflammatory cascade in response to Aβ and tau formation, which in turn cyclically increases their production [3, 4]. Meanwhile, relatively little is known about how immunoglobulins, that constantly evolve with exposure to antigens, may be associated with early biomarkers of dementia.

Immunoglobulins, including IgA, IgG, and IgM, are produced by B-lymphocytes in response to different stimuli [5]. The role of these immunoglobulins in the pathophysiology of dementia remains uncertain, as studies thus far have reported conflicting results [6–8]. In our previous work, we did not find an association between serum immunoglobulins and prevalent or incident dementia, but noted that individuals with poor cognition exhibited higher levels of serum IgG [9]. Consistent with the latter observation, several studies have reported high levels of immunoglobulins targeting proteins in nerve cells, such as the N-Methyl-D-Asparate receptor (NMDAR) [10–12], which suggest that immunoglobulins may have detrimental effects on synaptic transmission and plasticity. However, certain immunoglobulins have also been attributed protective effects, as they are inherently produced in response to Aβ and tau formation [7, 13]. For instance, cognitively healthy older adults are known to exhibit higher levels of IgM targeting Aβ compared to individuals with Alzheimer’s disease [8]. Such observations prompted numerous therapeutic trials to explore the use of intravenous immunoglobulins (IVIG) for Alzheimer’s disease [14], but with moderate success. While research on antigen-specific immunoglobulins is advancing, studies using general inflammation markers have provided additional insights. In a prior population-based study, a higher lymphocyte count was suggested to be neuroprotective, while elevated granulocyte and platelet levels were associated with an increased risk of dementia [15]. In the current study, we raised the hypothesis that immunoglobulin levels are associated with early markers of dementia, including neurofilament light chain (NfL), t-tau, and Aβ, as well as structural markers on neuroimaging. This hypothesis is based on the premise that inflammatory processes can disrupt neuronal integrity [16]. To date, prior studies were underpowered or only focused on specific antigens while examining such associations [6, 11, 17].

To advance insight in pathways underlying inflammation and dementia, we determined cross-sectional associations between whole serum immunoglobulin (IgA, IgG and IgM) levels and plasma biomarkers of dementia, including Aβ, t-tau and NfL, in a large population-based cohort. These plasma biomarkers have recently been introduced as non-invasive indicators of neuroaxonal damage (NfL, t-tau) and the accumulation of amyloid beta (Aβ-40, Aβ-42) [18]. Likewise, by studying associations between immunoglobulins and structural neuroimaging markers, such as brain atrophy and white matter disintegrity, we attempt to identify neuropathological correlates of an inflammatory state.

## Materials and methods

### Study population

This study was conducted within the Rotterdam Study: a large, prospective, population-based cohort of the Netherlands, details of which have been published previously [19]. In brief, the Rotterdam Study initiated in 1990 when all inhabitants of Ommoord (a suburb of Rotterdam) aged ≥ 55 years were invited to participate (enrolled *n* = 7983, RS-I). This cohort was subsequently expanded thrice: first in 2000, with 3,011 individuals who had reached the eligible age or had moved into the study area (RS-II), later in 2006, with 3,932 individuals from the same area aged 45 or over (RS-III) and last in 2015 with 3,005 individuals from the same area aged 40 or over (RS-IV). Participants engage in comprehensive interviews and are examined at a research facility every 3–6 years. Subsequently, participants are continuously monitored though electronic linkage of medical records with the study database.

The present study includes individuals for whom serum immunoglobulins (IgA, IgG and IgM) were measured at study entry, originating from the third examination round of RS-I and the first examination rounds of RS-II and RS-III. Out of 8,768 eligible participants with informed consent and serum immunoglobulins available, we only included individuals who also had 1) plasma biomarkers of dementia (NfL, t-tau, Aβ-40 and Aβ-42) or 2) a brain MRI, performed within a time-interval of maximum 5 years after measurement of immunoglobulins.

### Assessment of serum immunoglobulins

Blood samples collected at study entry were stored in ethylenediamine tetra-acetic acid (EDTA)-treated containers and frozen at -80 °C, according to standard procedures. From 2016 to 2018, these samples were thawed for analytic purposes. An immunoturbidimetric assay (Tina-quant® IgA/IgG/IgM Gen. 2, Roche Diagnostics GmbH, Mannheim, Germany) was used to measure serum IgA, IgG, and IgM levels, yielding coefficients of variation of 1.12–2.68% between batches (effect of time) and 2.05–3.58% across batches (assay precision) for each immunoglobulin. The manufacturer’s protocol provided reference ranges of serum immunoglobulins for adults, which were 0.7–4.0 g/L for IgA, 7.0–16.0 g/L for IgG, and 0.4–2.3 g/L for IgM. Reference ranges for our study population, based on the 2.5th-97.5th percentiles (in accordance with practice in clinical chemistry) [20] were: 0.86–4.76 g/L for IgA, 6.20–15.10 g/L for IgG, and 0.28–2.64 g/L for IgM.

### Assessment of NfL, t-tau, Aβ-40 and Aβ-42

Blood collected from participants was stored in EDTA-containers and then centrifuged. According to standard procedures, plasma was aliquoted and frozen at − 80 °C. The Quanterix assay (Lexington, MA,USA) was used to analyse samples in two batches, using a single molecule array (Simoa) HD-1 analyzer platform [21]. The NF-light advantage kit [22] was used to assess levels of NfL and the Simoa Human Neurology 3-Plex assay was used to measure levels of Aβ-40, Aβ-42 and t-tau. Samples were tested twice and on each plate, two quality control (QC) samples were run for each biomarker. One control consisted of an antigen spiked in control buffer, and the other control involved a positive plasma control pool containing an endogenous antigen. Pre-specified nominal values and acceptance ranges were established for each antigen control, as described previously [23], where extended details on assay performance can be found. Data was excluded from analysis in case of: 1) duplicates, 2) missing measurements, 3) concentration coefficient of variation exceeding 20% or 4) out of range samples in controls.

### Neuroimaging protocol

A multi-sequence magnetic resonance imaging (MRI) protocol was implemented in the Rotterdam Study from 2005 and onwards, performed on a single 1.5 Tesla scanner (General Electric Healthcare, Milwaukee, WI) with a 8-channel head coil and comprising amongst others of a high-resolution axial T1-weighted sequence, a fluid-attenuated inversion recovery sequence, a proton density–weighted sequence, and a T2*-weighted gradient echo sequence [24]. A k-nearest neighbor tissue classification algorithm [25] was applied for image pre-processing and estimation of total brain volume (TBV), gray matter (GM), normal-appearing white matter (WM) and white matter hyperintensities (WMH). Hippocampal volumes (HV) were determined using FreeSurfer 6.1 [26]. All segmentations were inspected by trained researchers and manually corrected if needed.

From 2006 onwards, a diffusion-weighted imaging sequence was added to the scan protocol [27]. Pre-processing steps included eddy current, head motion correction and fitting of diffusion tensors. Fractional anisotropy (FA) and mean diffusivity (MD) were calculated in the normal-appearing white matter. Lower FA and higher MD values suggest worse structural white matter integrity.

### Covariates

Information on educational attainment, current smoking habits, alcohol consumption, medical history and medication use was acquired from home interviews and supplemented with medical records. Educational attainment was categorized according to the UNESCO International Standard Classification of Education (United Nations Educational 1976) as follows: primary education (primary), lower/intermediate general education, lower vocational education, intermediate vocational education or higher general education (further), and higher vocational education or university (higher). Smoking habits were categorized into never, former or current smoker. Based on answers from questionnaires, alcohol consumption was estimated in grams/day and categorized into none, mild (0–10 g/day), moderate (10–20 g/day), and heavy (> 20 g/day). History of stroke or coronary heart disease (CHD) was self-reported during home interviews and verified using medical records that were linked to the study database. A participant with a history of myocardial infarction, percutaneous coronary intervention or coronary artery bypass grafting was classified with CHD. Serum creatinine levels were determined using an enzymatic assay method and reported in micromoles per liter (µmol/L), after which the estimated glomerular filtration rate (eGFR) was calculated using the Chronic Kidney Disease Epidemiology Collaboration (CKD‐EPI) formula [28]. Data on medication that may influence serum immunoglobulin levels (systemic corticosteroids, antiepileptic drugs, angiotensin converting enzyme inhibitors, cytostatics, immunomodulators, and/or immunosuppressants) was also collected at home-interviews.

During center visits, blood pressure was measured twice in a sitting position, using a random-zero sphygmomanometer on the right arm. The mean of two measurements was used to assess the presence of hypertension, defined as a blood pressure of ≥ 140/90 mmHg and/or the use of blood pressure lowering drugs. Body mass index (BMI) was computed as weight in kilograms divided by height in meters squared. Blood samples taken during center visits provided information on serum total cholesterol, high-density lipoprotein cholesterol and glucose levels. Hypercholesterolemia was regarded as a total cholesterol value of ≥ 6.2 mmol/L in serum or use of lipid-lowering medication. Type 2 diabetes was defined as a fasting serum glucose level ≥ 7.0 mmol/L (126 mg/dL), a nonfasting serum glucose level ≥ 11.1 mmol/L (200 mg/dL), and/or the use of blood glucose-lowering medication. *APOE* genotype was determined using polymerase chain reaction on coded DNA samples (RS-I) and biallelic Taqman assays (RS-II and RS-III) (TaqMan Gene Expression Assays; Thermo Fisher Scientific, Waltham, Massachusetts) (rs7412 and rs429358). For the analysis, carriers and non-carriers of the *APOE*-ε4 allele were distinguished based on *APOE* genotype, wherein participants ε2/ε4, ε3/ε4 and ε4/ε4 genotypes were classified as carriers and ε2/ε2, ε2/ε3 and ε3/ε3 genotypes were classified as non-carriers.

### Statistical analysis

Cross-sectional associations between serum immunoglobulins and plasma biomarkers of dementia (NfL, t-tau, Aβ-40 and Aβ-42) were explored with linear regression models, from which we obtained mean differences and corresponding 95% confidence intervals (95% CIs). Potential confounders were identified by literature review and based on biological plausibility [29, 30]. Model I was adjusted for age at immunoglobulin assessment, sex, cohort and time-interval between immunoglobulin and biomarker assessments. Model II was additionally adjusted for *APOE*-ε4 carriership, educational attainment, alcohol consumption and smoking status. Model III was additionally adjusted for BMI, hypertension, estimated glomerular filtration rate, hypercholesterolemia, diabetes mellitus, history of CHD and history of stroke. For cross-sectional associations between serum immunoglobulins and neuroimaging markers, we used a similar set of models and potential confounders as described earlier, with additional adjustment for intracranial volume. Models of WMH, FA and MD were also corrected for normal appearing white matter volume.

Measurements in blood, such as serum immunoglobulins, may to some extent depend on population characteristics, medication use and temporary inflammatory conditions. Therefore, we performed a sensitivity analysis excluding participants with immunoglobulin levels beyond the reference range (based on percentiles of this population) and use of medication that may influence serum immunoglobulins. Furthermore, we performed two stratified analyses, where we studied associations separately for 1) carriers and non-carriers of the *APOE*-ε4 allele and 2) men and women. The considerations for stratified analyses were based on the knowledge that the *APOE*-ε4 allele modifies both the central and systemic inflammatory response [31], and that serum immunoglobulin levels may be different between men and women [29].

Prior to running the analysis, plasma concentrations (pg/ml) of NfL, t-tau, Aβ-40 and Aβ-42 were log_2_ transformed to achieve normal distributions. WMH volume (cm^3^) was natural log-transformed, due to its left-skewed distribution. Serum immunoglobulins, plasma NfL, t-tau, Aβ-40 and Aβ-42 and all neuroimaging markers were then standardized to facilitate comparison. Standardization involved centering around the mean with the standard deviation representing one unit (z-score normalization). Missing data on covariates (maximum 9.3%) were imputed using fivefold multiple imputation and similar distributions were observed before and after imputation. Multiple testing correction was applied using the false discovery rate (FDR) of Benjamini–Hochberg to the main biomarker and neuroimaging models. For sensitivity and stratified analyses, a suggestive association was considered at a nominal significance threshold of α = 0.05 (*P* < 0.05). Analyses were performed using R version 3.6.1 (packages mice, tidyr, dplyr, lubridate, foreign) and IBM SPSS Statistics version 24.0 (IBM Corp, Somers, NY).

## Results

From 8,768 participants who gave informed consent and had serum immunoglobulins available, 3,455 had assessments of plasma biomarkers of dementia and 3,139 visited the centre for a brain MRI within five years after blood sampling (median time-interval in years 0.07, interquartile range (IQR) [0.04 – 0.15]). Characteristics of the study population are presented in Table 1. The median age of participants with plasma biomarkers was 65.8 years [IQR 61.5 – 72.0], and 57.2% were female. The median age of participants with neuroimaging data was 57.4 years [IQR 52.7 – 60.7], and 54.4% were female. Only 285 participants were overlapping between the two study samples and the number of participants with prevalent dementia was limited (*n* = 3). Throughout the results section, we present the results of model II, which includes the main potential confounders. Similar results were obtained for model I (the basic model: adjusted for age, sex, cohort and time difference) and model III (adjusted for covariates that may act as confounders or mediators), details of which can be found in the Supplementary files.

**Table 1:** Characteristics of the study population

**Characteristic**	**Sample with plasma biomarkers of dementia (*N* = 3455)**	**Sample with neuroimaging data (*N* = 3139)**
Sex (%)		
Female	1976 (57.2)	1709 (54.4)
Age in years [median, IQR]	65.8 [61.5, 72.0]	57.4 [52.7, 60.7]
Educational attainment (%)		
Primary education	334 (9.8)	308 (9.9)
Further education	2565 (75.0)	1994 (63.8)
Higher education	519 ( 15.2)	823 (26.3)
*APOE*-ε4 carrier (%)	920 (27.4)	875 (29.8)
Smoking status (%)		
Never	1052 (30.8)	923 (29.5)
Former	1830 (53.5)	1571 (50.2)
Current	537 (15.7)	635 (20.3)
Alcohol consumption (%)		
None	474 (21.3)	315 (11.1)
Mild (0–10 g/day)	961 (43.2)	1823 (64.1)
Moderate (10–20 g/day)	357 (16.1)	530 (18.6)
Heavy (> 20 g/day)	430 (19.4)	178 (6.3)
Hypertension (%)	1271 (37.8)	1137 (36.6)
Hypercholesterolemia (%)	1544 (44.7)	1437 (45.9)
Body mass index in kg/m^2^ (mean, SD)	27.0 (3.9)	27.4 (4.3)
Estimated glomerular filtration rate (mean, SD)	87.3 (14.4)	76.8 (14.0)
Diabetes (%)	380 (11.0)	290 (9.2)
History of stroke (%)	90 (2.6)	68 (2.2)
History of coronary heart disease (%)	233 (6.8)	114 (3.6)
Use of drugs affecting immunoglobulins (%)	420 (12.4)	468 (15.0)
Mini Mental State Examination (MMSE) score [median, IQR]	28.0 [27.0, 29.0]	28.0 [27.0, 29.0]
Serum IgA in g/L [median, IQR]	2.1 [1.6, 2.7]	2.0 [1.5, 2.7]
Serum IgG in g/L [median, IQR]	9.5 [8.1, 10.9]	9.8 [8.5, 11.2]
Serum IgM in g/L [median, IQR]	0.9 [0.6, 1.2]	0.9 [0.6, 1.2]
Plasma neurofilament light chain (pg/mL)	15.3 (12.0)	*14.0 (14.4)*
Plasma total tau (pg/mL)	2.6 (2.5)	*2.6 (0.9)*
Plasma amyloid-beta 40 (pg/mL)	262.7 (53.3)	*255.1 (46.3)*
Plasma amyloid-beta 42 (pg/mL)	10.5 (3.0)	*11.1 (5.5)*
Total intracranial volume in cm^3^ (mean, SD)	*1151.0 (114.3)*	1144.1 (117.0)
Total brain volume in cm^3^ (mean, SD)	*936.4 (95.7)*	958.9 (99.4)
Gray matter volume in cm^3^ (mean, SD)	*528.6 (48.9)*	538.8 (54.9)
White matter volume in cm^3^ (mean, SD)	*407.8 (59.6)*	420.1 (58.7)
White matter lesions in cm^3^ [median, IQR]	*3.7 [2.3, 7.1]*	2.2 [1.4, 3.9]
Hippocampal volume in cm^3^ (mean, SD)	*6.7 (0.7)*	6.9 (0.7)
Fractional anisotropy (mean, SD)	-	0.34 (0.02)
Mean diffusivity (mean, SD)	-	0.73 (0.02)

N = number of participants included in the study. Only 285 participants were overlapping between both study samples, for whom characteristics are *displayed in italic*. Data presented are not imputed. Missing covariate data for the biomarker sample: alcohol (3.6%), *APOE*-ε4 carriership (3.0%), hypertension (2.6%), use of drugs affecting immunoglobulins (2.0%), education (1.1%), history of coronary heart disease (0.2%), smoking (1.0%), body mass index (0.9%). Missing covariate data for the MRI sample: alcohol (9.3%), *APOE*-ε4 carriership (6.3%), hypertension (1.0%), use of drugs affecting immunoglobulins (0.8%), education (0.4%), smoking (0.3%), hypercholesterolemia (0.2%), history of coronary heart disease (0.2%), body mass index (0.1%)

*Abbreviations*: *IgA* immunoglobulin A, *IgG* immunoglobulin G, *IgM* immunoglobulin M, *SD* standard deviation, *IQR* interquartile range, *g* gram, *pg* pictogram, *mL* milliliter, *L* liter, *cm* centimeters

### Main analysis: immunoglobulins and biomarkers of dementia

**Fig. 1:**
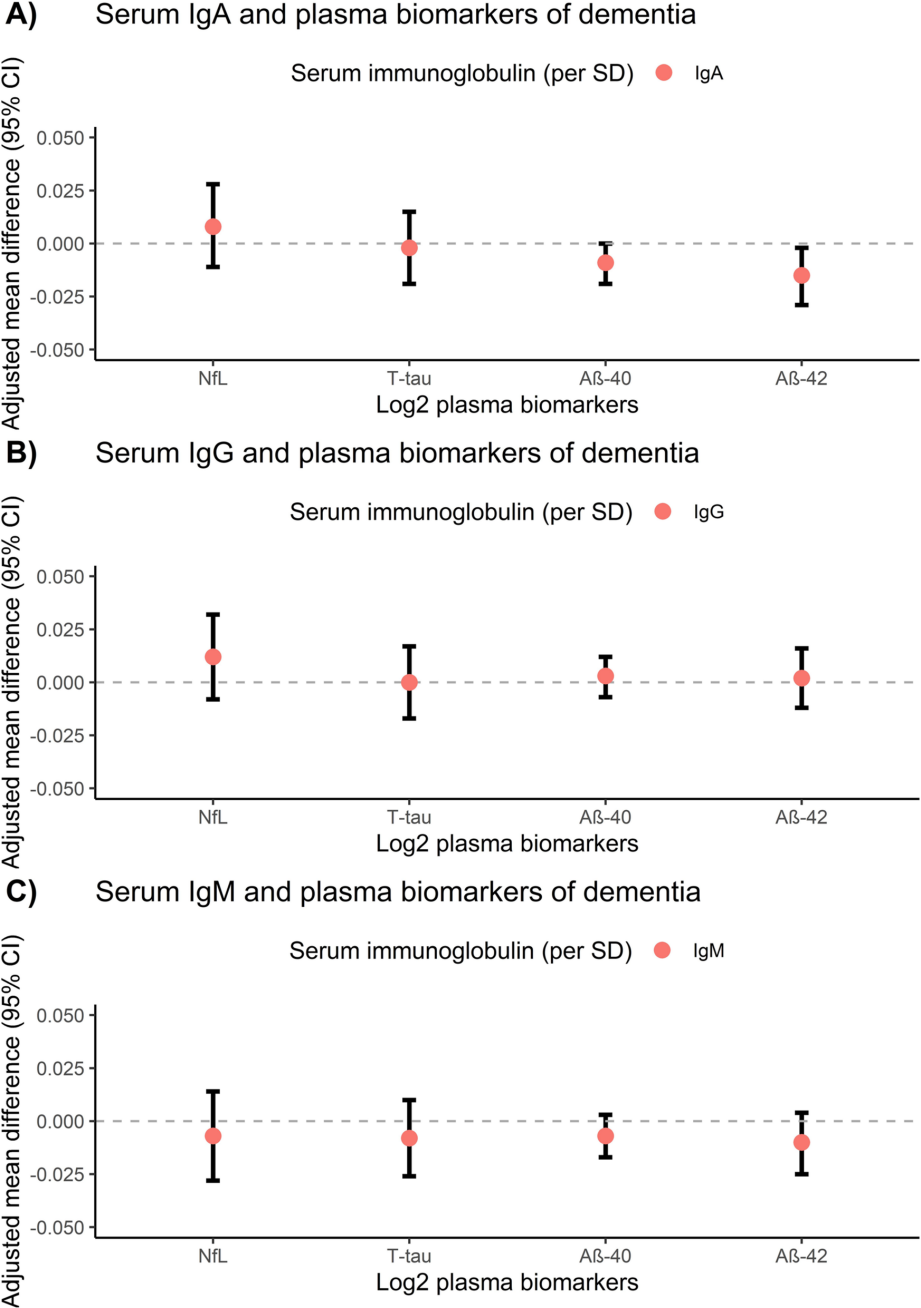
Overall associations between serum immunoglobulins and plasma biomarkers of dementia. Associations are presented as adjusted mean differences (95% confidence interval) for 3455 study participants. Model II is displayed, which is adjusted for age, sex, study cohort, time difference, smoking status, alcohol consumption, educational attainment and *APOE*-ε4 carriership. Abbreviations: NfL = neurofilament light chain, t-tau = total tau, SD = standard deviation, CI = confidence interval. No associations reached statistical significance, after accounting for multiple testing using the false discovery rate. A detailed overview of all models (I-III) can be found in Supplementary Table 1

**Fig. 2:**
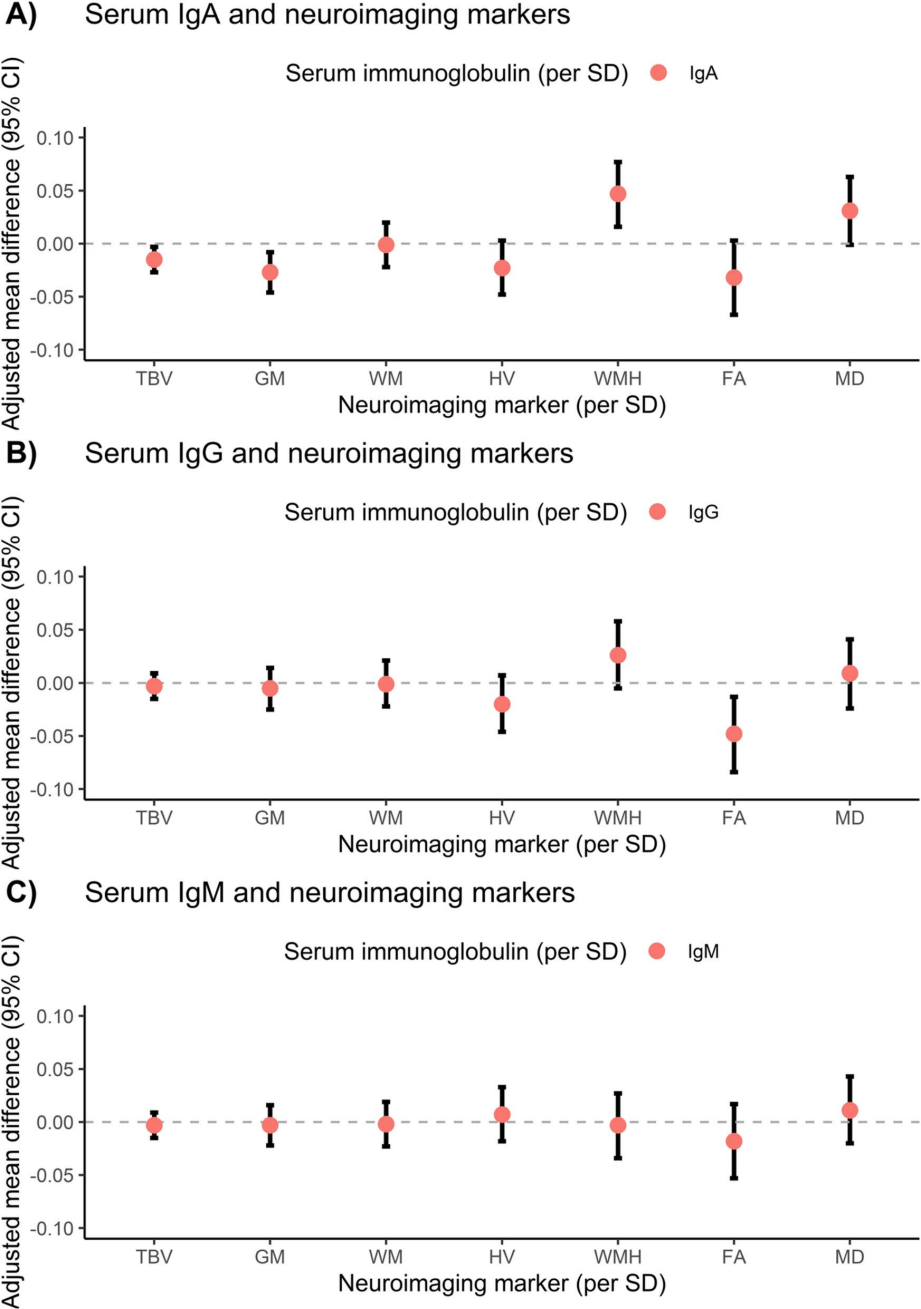
Associations between serum immunoglobulins and neuroimaging markers. Associations are presented as adjusted mean differences (95% confidence interval) for 3139 study participants. Model II is displayed, which is adjusted for age, sex, cohort, intracranial volume, time difference, smoking status, alcohol consumption, educational attainment and *APOE*-ε4 carriership. All neuroimaging markers and serum immunoglobulins were standardized. Models of WMH, FA and MD were additionally corrected for normal appearing white matter volume. Abbreviations: TBV = total brain volume, GM = gray matter volume, WM = white matter volume, HV = hippocampal volume, WMH = white matter hyperintensities, FA = fractional anisotropy, MD = mean diffusivity, SD = standard deviation, CI = confidence interval. No associations reached statistical significance, after accounting for multiple testing. A detailed overview of all models (I-III) can be found in Supplementary Table 2

After applying multiple testing correction, none of the associations between serum immunoglobulins and biomarkers of dementia, either in plasma or neuroimaging, reached statistical significance. However, several suggestive associations emerged in the main analysis (Figs. 1 and 2): participants with higher levels of IgA tended to have lower plasma levels of Aβ-42 (standardized adjusted mean difference (model II): -0.015 [95% CI: -0.029−-0.002], *p* = 0.028, Supplementary Table 1). In addition, they had more atrophy of total brain (model II: -0.015 [95% CI: -0.027−-0.003], *p* = 0.012, Supplementary Table 2), which was primarily driven by gray matter loss (model II: -0.027 [95% CI: -0.046−-0.008], *p* = 0.006), and a higher volume of white matter hyperintensities (model II: 0.047 [95% CI: 0.016−0.077], *p* = 0.003). No associations were observed between serum IgG levels and plasma biomarkers of dementia, although higher levels of IgG concurred with reduced fractional anisotropy (model II: -0.048 (-0.084−-0.013), *p* = 0.007, Supplementary Table 2). No associations were found between serum IgM and markers of neurodegeneration, either in plasma or on brain MRI.

### Sensitivity analysis

**Fig. 3:**
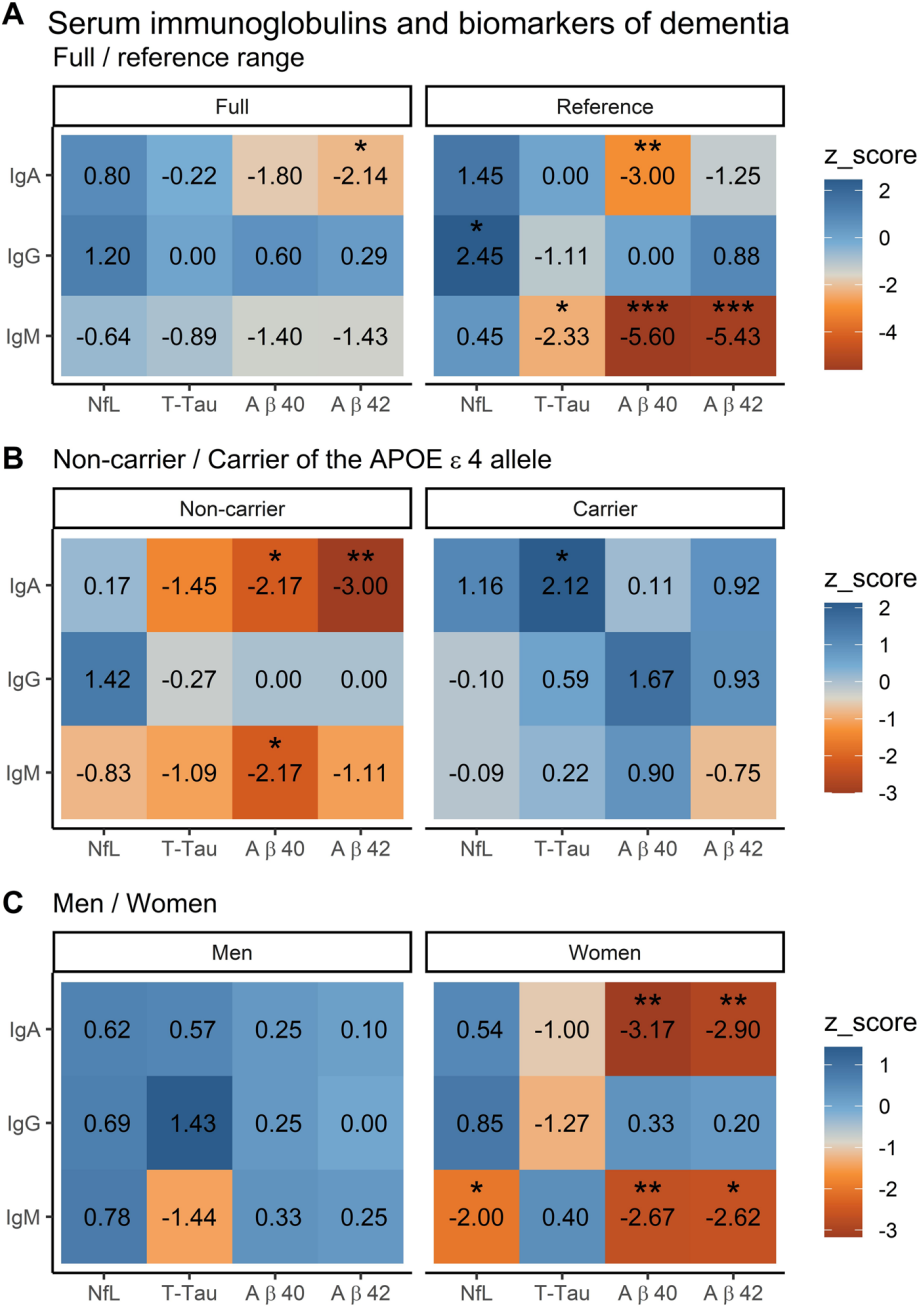
Sensitivity and stratified analyses: associations between serum immunoglobulins and plasma biomarkers of dementia. Associations are presented using z-scores (adjusted mean difference (β)/standard error (SE)) for 3455 study participants. Higher z-scores represent positive associations, whereas lower z-scores represent negative associations. Model II is displayed, which is adjusted for age, sex, study cohort, time difference, smoking status, alcohol consumption, educational attainment and *APOE*-ε4 carriership, while excluding the variable that was stratified on. Abbreviations: NfL = neurofilament light chain, t-tau = total tau, Aβ = amyloid beta. Stars denote suggestive associations: * = *p* < 0.05, ** = *p* < 0.01, *** = *p* < 0.001. A detailed overview of all models (I-III) can be found in Supplementary Tables 3, 5 and 7.

**Fig. 4:**
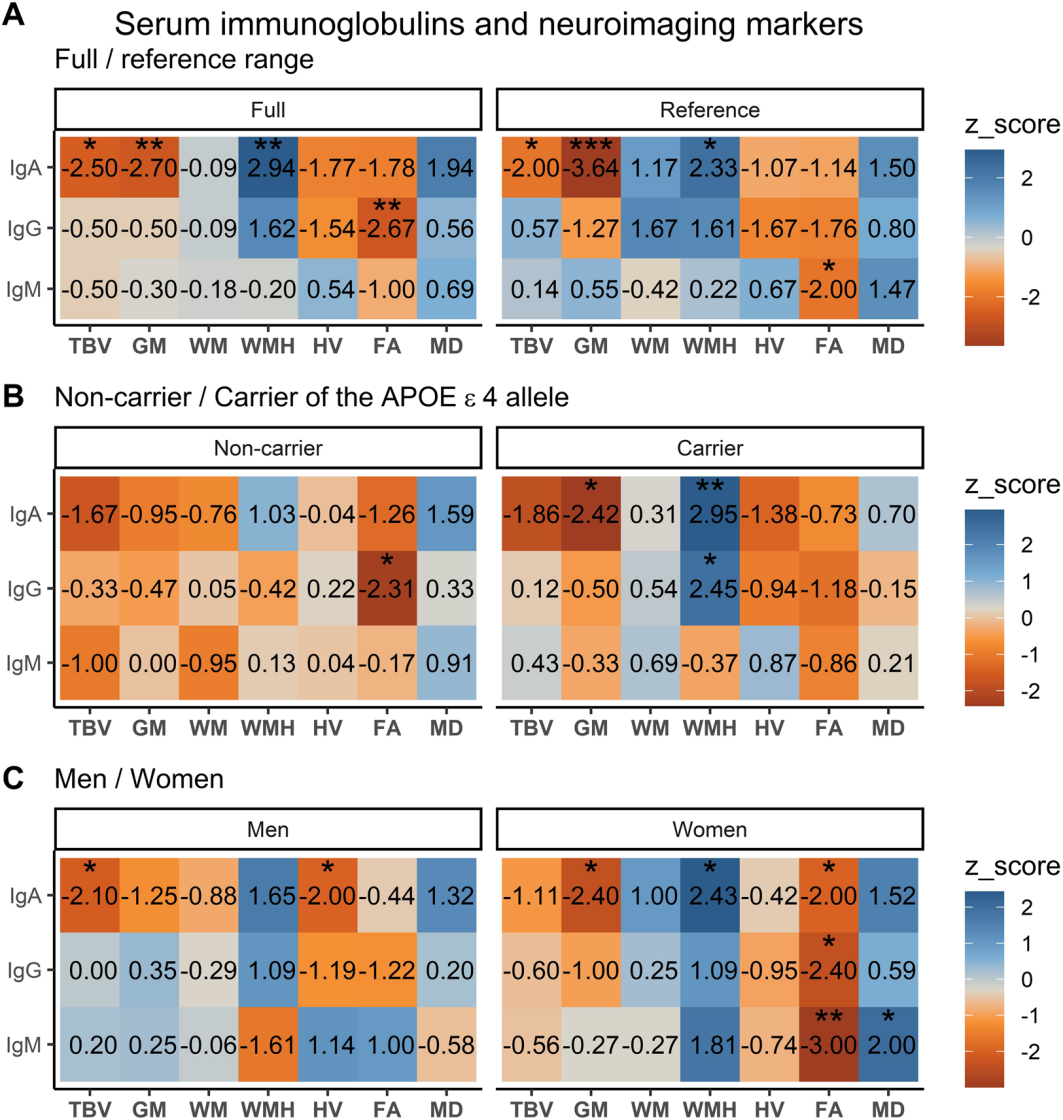
Sensitivity and stratified analyses: associations between serum immunoglobulins and neuroimaging markers. Associations are presented using z-scores (adjusted mean difference (β)/standard error (SE)) for 3139 study participants. Higher z-scores represent positive associations, whereas lower z-scores represent negative associations. Model II is displayed, which is adjusted for age, sex, cohort, intracranial volume, time difference, smoking status, alcohol consumption, educational attainment and *APOE*-ε4 carriership, while excluding the variable that was stratified on. All neuroimaging markers and serum immunoglobulins were standardized. Models of WMH, FA and MD were additionally corrected for normal appearing white matter volume. Abbreviations: TBV = total brain volume, GM = gray matter volume, WM = white matter volume, HV = hippocampal volume, WMH = white matter hyperintensities, FA = fractional anisotropy, MD = mean diffusivity. Stars denote suggestive associations: * = *p* < 0.05, ** = *p* < 0.01, *** = *p* < 0.001. A detailed overview of all models (I-III) can be found in Supplementary Tables 4, 6, and 8.

In sensitivity analyses, where we restricted to reference range of serum immunoglobulins and excluded participants using immunomodulating drugs, the direction of associations of IgA with Aβ-40 and Aβ-42 remained consistent to the main analysis, but statistical significance was only obtained for Aβ-40 (model II: -0.015 [95% CI: -0.025−-0.004], *p* = 0.005, Fig. 3A, Supplementary Table 3). Associations between IgA and neuroimaging markers were similar to those observed in the main analysis (Fig. 4A, Supplementary Table 4). While IgG did not relate to any of the plasma biomarkers in the main analysis, a suggestive association was found in the sensitivity analysis with NfL (model II: 0.027 [95% CI: 0.006—0.049], *p* = 0.013), yet associations with neuroimaging markers remained absent. In contrast to the main analysis, several suggestive associations were noted between IgM and t-tau (model II: -0.021 [95% CI: -0.040−-0.003], *p* = 0.023), Aβ-40 (model II: -0.028 [95% CI: -0.038−-0.018], *p* = 4.67 × 10^–8^), Aβ-42 (model II: -0.038 [95% CI: -0.052−-0.023], *p* = 3.21 × 10^–7^, Fig. 3A, Supplementary Table 3), as well as reduced fractional anisotropy on brain MRI (model II: -0.042 [95% CI: -0.082−-0.002], *p* = 0.041, Fig. 4A, Supplementary Table 4).

### Stratified analysis

Stratified analyses showed several patterns, such as that non-carriers of the *APOE*-ε4 allele with higher levels of IgA exhibited lower Aβ-40 and Aβ-42 (Fig. 3B, Supplementary Table 5). In contrast, *APOE*-ε4 carriers with elevated IgA levels exhibited higher t-tau, along with more gray matter atrophy and white matter hyperintensities (Fig. 4B, Supplementary Table 6). Stratified analyses for IgG did not reveal clear patterns regarding plasma biomarkers, but non-carriers of *APOE*-ε4 with higher IgG levels had lower fractional anisotropy on neuroimaging, while carriers had a higher volume of white matter hyperintensities (Fig. 4B, Supplementary Table 6). Regarding IgM, only non-carriers of *APOE*-ε4 had lower Aβ-40, while no patterns could be discerned for neuroimaging markers. In sex-stratified analyses, women with elevated IgA and IgM levels exhibited lower Aβ-40 and Aβ-42. Furthermore, higher IgM levels were associated with decreased NfL in women (Fig. 3C, Supplementary Table 7). On neuroimaging, women exhibited associations that closely resembled those of the main analysis, but with more compromised white matter microstructural integrity. Men did not display any associations of immunoglobulins with plasma biomarkers, although with higher IgA levels they exhibited more atrophy of total brain and the hippocampus (Fig. 4C, Supplementary Table 8).

## Discussion

After multiple testing correction, we could not confirm associations between serum immunoglobulins (IgA, IgG, IgM) and biomarkers of dementia in plasma or on neuroimaging. Nevertheless, several observations were made in sensitivity and stratified analyses that can be explored in future research. Among participants with immunoglobulin levels falling within the reference range and not using immunomodulating drugs, those with higher serum IgA levels had lower plasma Aβ-40, along with a smaller total brain volume (driven by gray matter loss) and a higher volume of white matter hyperintensities. Conversely, those with higher IgG levels had higher plasma NfL, indicating more neuroaxonal injury. In addition, participants with higher IgM levels exhibited lower levels of t-tau, Aβ-40, and Aβ-42 alike, but also had less white matter structural integrity on brain MRI. Our stratified analyses suggest that these patterns may differ for non-carriers and carriers of the *APOE*-ε4 allele, as well as for women and men. Further research is required for in-depth exploration of these differences.

The absence of consistent associations between serum immunoglobulins and biomarkers of dementia aligns with our previous research that investigated the link between serum immunoglobulins, cognition, and dementia [9]. Dementia is a complex syndrome with diverse underlying mechanisms, wherein serum immunoglobulins may perhaps not directly impact the specific biomarkers examined. Genetic variations in immune response [32], interactions with environmental exposures [29], or comorbidities not accounted for in this analysis, could also have contributed to the lack of strong associations. In addition, while neuroinflammation and neurodegeneration are both complex processes with widespread effects, their manifestation may only be partially captured by peripheral blood measures of immunoglobulins (in serum) and biomarkers of dementia (in plasma).

Overall, this study illustrates the intricate relationship between immunoglobulins and early biomarkers of dementia. IgA, an immunoglobulin abundant in mucous membranes, was previously shown to be elevated in saliva of patients with mild Alzheimer’s disease, but not in those with moderate to severe Alzheimer’s disease [17]. This phenomenon may be best explained by hyperactivity of the immune response in early stages of Alzheimer’s disease, possibly resulting in an enhanced clearance of Aβ in plasma, which may later transition to lower but chronic activation of the adaptive immune system as neurodegeneration ensues [33]. In both the overall and reference population, we observed that higher serum IgA levels concurred with lower plasma levels of Aβ, which could either indicate appropriate clearance mechanisms at an early stage, or more sequestration of Aβ in the brain. What supports the latter view is that in neuroimaging associations, higher levels of IgA were seen with a smaller total brain volume, particularly gray matter, and a higher volume of white matter hyperintensities. However, it is also worth noting that serum IgA interacts with many other serum components, such as albumin, a1-antitrypsin, the HC-protein and fibronectin, and is influenced by the mucosa [34]. Therefore, the values of IgA may be relatively low in serum and not comparable to for instance, saliva.

Given the abundance of IgG in serum and its involvement in the secondary immune response, we postulated that it would be elevated during chronic neuroinflammatory processes. Within this context, high IgG levels could signify a disruption of the blood–brain-barrier [35]. Several studies have previously shown that high levels of IgG are found in serum, cerebrospinal fluids and brains of post-mortem patients with Alzheimer’s disease [36, 37]. Although we could not establish this association within our overall study population, we did observe a suggestive association amongst individuals with immunoglobulin levels within the reference range and those who were not using immunomodulating drugs (sensitivity analysis). In this particular subgroup, higher IgG levels concurred with higher NfL, suggesting greater neuroaxonal damage, but this relationship did not extend to neuroimaging markers. Notably, our study cohort primarily consisted of individuals with subtle signs of neurodegeneration in plasma and brain MRI, but without clinical dementia, which could also account for the lack of associations.

IgM is the first immunoglobulin to be produced during B-cell ontogeny, followed by class-switching to IgG or formation of T-cell memory cells. Due to their pentameric structure, IgM antibodies have a high avidity for binding to antigens, such as Aβ or tau [38], which can facilitate their clearance. In our study, this did not apply to the entire study population, but only to participants with immunoglobulin levels in the reference range and not using immunomodulating drugs. This subset of participants exhibited higher serum IgM levels and lower plasma levels of t-tau, Aβ-40 and Aβ-42, and diminished white matter microstructural integrity. In keeping with our observations, a previous study reported significantly higher levels of plasma IgM targeted against Aβ-42 in healthy aged subjects, compared to young controls [8]. Conversely, in AD subjects compared to age-matched controls, they observed a decline of IgM targeted against Aβ-42. Taken together, these findings imply that immunocompetence for IgM could be important in early pathological stages of Alzheimer’s disease [6], but that this effectiveness appears to wane as neurodegeneration advances or when dementia manifests.

Consistent with associations described for innate immunity in previous literature [4, 15, 31], carriership of the *APOE*-ε4 allele may play an important role in the association between serum immunoglobulins and plasma biomarkers of dementia. In the brain, *APOE* is predominantly expressed by astrocytes and microglia. Carriership of the *APOE*-ε4 allele has been shown to disrupt homeostasis of these glial cells [39], prompting an enhanced inflammatory cascade in response to antigens (such as Aβ-40 and Aβ-42), which could have implications for immunoglobulins. Indeed, with elevated IgA levels, our stratified analyses suggest lower plasma Aβ in non-carriers of the *APOE*-ε4 allele, while *APOE*-ε4 carriers show more signs of neuronal compromise, including higher t-tau levels, increased gray matter atrophy, and more white matter hyperintensities.

Sex-differences have long been acknowledged for inflammatory neurodegenerative disorders, such as multiple sclerosis [40]. Previous research has shown that in post-mortem brains of patients with Alzheimer’s disease, women tend to have a higher Aβ burden compared to men [41]. Such differential patterns were also observed in our sex-stratified analyses. Among women with elevated IgA and IgM levels, we found lower plasma Aβ levels. However, in women with higher IgA levels, we also noted increased gray matter atrophy, more white matter hyperintensities, and compromised white matter integrity. In contrast, men with higher IgA levels showed no associations with biomarkers in plasma, but predominantly had total brain and hippocampal atrophy.

### Strengths and limitations

Strengths of this study include a population-based study design, involving a large sample of individuals with standardized assessment of biomarkers, both in plasma and on neuroimaging. Several limitations of this study should be acknowledged. First, associations were determined cross-sectionally, making it difficult to establish the temporality of the observed associations. Longitudinal cohort studies may advance our understanding of how changes in serum immunoglobulins over time affect biomarkers of dementia. Second, previously described associations with antigen-specific (Aβ/tau) immunoglobulins and overall immunoglobulin levels used in this study may differ, as the latter is elevated in response to a large variety of inflammatory processes. However, by studying overall immunoglobulin levels, we may get a more comprehensive overview, while also accounting for peripheral inflammation. Third, since phosphotau was not available, we used plasma NfL and total tau as biomarkers for dementia, which both reflect neuroaxonal damage rather than Alzheimer-related pathology. Fourth, due to the limited number of persons with dementia in our study population (*n* = 3), we were not able to study associations in advanced neuropathology. Lastly, the results from our predominantly White population may not generalize to other ethnicities, who may have different inflammatory profiles [42].

## Conclusion

In this population-based sample, we could not confirm associations between serum immunoglobulins and early pathological markers of dementia. Nevertheless, our exploratory analyses suggest that sex and carriership of the *APOE*-ε4 allele may be important variables that need to be factored into this relationship. Future research is warranted to validate these findings and elucidate the role of these factors in relation to immunoglobulins and biomarkers of dementia.

### Supplementary Information

**Additional file 1: Supplementary Table 1.** Associations between serum immunoglobulins and plasma biomarkers of dementia. **Supplementary Table 2.** Associations between serum immunoglobulins and neuroimaging markers. **Supplementary Table 3.** Associations between serum immunoglobulins and plasma biomarkers of dementia, full compared to reference range. **Supplementary Table 4.** Associations between serum immunoglobulins and neuroimaging markers, full compared to reference range. **Supplementary Table 5.** Associations between serum immunoglobulins and plasma biomarkers of dementia, stratified by *APOE*-ε4 carriership. **Supplementary Table 6.** Associations between serum immunoglobulins and neuroimaging markers, stratified by *APOE*-ε4 carriership. **Supplementary Table 7.** Associations between serum immunoglobulins and plasma biomarkers of dementia, stratified by sex. **Supplementary Table 8.** Associations between serum immunoglobulins and neuroimaging markers, stratified by sex.

## Data Availability

Reasonable requests for data access may be considered by the management team of the Rotterdam Study (secretariat.epi@erasmusmc.nl). Given the privacy concerns, regulations and informed consent provided by participants, the data cannot be uploaded to a freely accessible public repository.

## Abbreviations

IgA: Immunoglobulin A
IgG: Immunoglobulin G
IgM: Immunoglobulin M
*APOE*: Apolipoprotein E
Aβ: Amyloid beta
NfL: Neurofilament light chain
t-tau: Total tau
TBV: Total brain volume
GM: Gray matter
WM: White matter
WMH: White matter hyperintensities
HV: Hippocampal volume
FA: Fractional anisotropy
MD: Mean diffusivity
