# Supplementary material for: Serum immunoglobulins and biomarkers of dementia: a population-based study
Source: Alzheimers Res Ther. 2023 Nov 7;15:194. doi: 10.1186/s13195-023-01333-3 (PMC10629143; doi:10.1186/s13195-023-01333-3)
Supplement: Supplementary file 1 — Additional file 1: Supplementary Table 1. Associations between serum immunoglobulins and plasma biomarkers of dementia. Supplementary Table 2. Associations between serum immunoglobulins and neuroimaging markers. Supplementary Table 3. Associations between serum immunoglobulins and plasma biomarkers of dementia, full compared to reference range. Supplementary Table 4. Associations between serum immunoglobulins and neuroimaging markers, full compared to reference range. Supplementary Table 5. Associations between serum immunoglobulins and plasma biomarkers of dementia, stratified by APOE-ε4 carriership. Supplementary Table 6. Associations between serum immunoglobulins and neuroimaging markers, stratified by APOE-ε4 carriership. Supplementary Table 7. Associations between serum immunoglobulins and plasma biomarkers of dementia, stratified by sex. Supplementary Table 8. Associations between serum immunoglobulins and neuroimaging markers, stratified by sex. [file 13195_2023_1333_MOESM1_ESM.docx]

**Additional file 1 to “Serum immunoglobulins and biomarkers of dementia: a population-based study”: Page:***Main analyses:*
Supplementary Table 1. Associations between serum immunoglobulins and plasma biomarkers of dementia 2
Supplementary Table 2. Associations between serum immunoglobulins and neuroimaging markers 3

*Sensitivity analyses:*
Supplementary Table 3. Associations between serum immunoglobulins and plasma biomarkers of dementia, full compared to reference range 4
Supplementary Table 4. Associations between serum immunoglobulins and neuroimaging markers, full compared to reference range 5

*Stratified analyses:*
Supplementary Table 5. Associations between serum immunoglobulins and plasma biomarkers of dementia, stratified by *APOE*-ε4 carriership 6
Supplementary Table 6. Associations between serum immunoglobulins and neuroimaging markers, stratified by *APOE*-ε4 carriership 7
Supplementary Table 7. Associations between serum immunoglobulins and plasma biomarkers of dementia, stratified by sex 8
Supplementary Table 8. Associations between serum immunoglobulins and neuroimaging markers, stratified by sex 9

**Supplementary Table 1. Associations between serum immunoglobulins and plasma biomarkers of dementia**

|  | Adjusted mean difference (95% CI) | | | |  |
| --- | --- | --- | --- | --- | --- |
|  | **Log2 NfL** | **Log2 T-tau** | **Log2 Aβ-40** | **Log2 Aβ-42** |  |
| Model I |  |  |  |  |  |
| IgA (per SD) | 0.008 (-0.011 - 0.028) | -0.003 (-0.020 - 0.014) | -0.010 (-0.019 - 0.000) | -0.014 (-0.028 - 0.000) |  |
| IgG (per SD) | 0.016 (-0.004 - 0.036) | -0.001 (-0.018 - 0.016) | 0.003 (-0.006 - 0.013) | 0.006 (-0.008 - 0.019) |  |
| IgM (per SD) | -0.007 (-0.028 - 0.013) | -0.009 (-0.027 - 0.009) | -0.008 (-0.017 - 0.002) | -0.010 (-0.024 - 0.004) |  |
| Model II |  |  |  |  |  |
| IgA (per SD) | 0.008 (-0.011 - 0.028) | -0.002 (-0.019 - 0.015) | -0.009 (-0.019 - 0.000) | -0.015 (-0.029 - -0.002) |  |
| IgG (per SD) | 0.012 (-0.008 - 0.032) | 0.000 (-0.017 - 0.017) | 0.003 (-0.007 - 0.012) | 0.002 (-0.012 - 0.016) |  |
| IgM (per SD) | -0.007 (-0.028 - 0.014) | -0.008 (-0.026 - 0.010) | -0.007 (-0.017 - 0.003) | -0.010 (-0.025 - 0.004) |  |
| Model III |  |  |  |  |  |
| IgA (per SD) | 0.013 (-0.005 - 0.032) | -0.003 (-0.020 - 0.013) | -0.009 (-0.018 - 0.000) | -0.012 (-0.025 - 0.001) |  |
| IgG (per SD) | 0.006 (-0.013 - 0.025) | -0.005 (-0.022 - 0.012) | -0.003 (-0.012 - 0.006) | -0.004 (-0.018 - 0.009) |  |
| IgM (per SD) | -0.011 (-0.031 - 0.008) | -0.008 (-0.025 - 0.010) | -0.009 (-0.018 - 0.001) | -0.012 (-0.026 - 0.002) |  |
| Associations are presented as adjusted mean differences (95% confidence interval) for 3455 study participants. Model I is adjusted for age, sex, study cohort and time difference. Model II is additionally adjusted for smoking status, alcohol consumption, educational attainment and APOE-ε4 carriership. Model III is additionally adjusted for body mass index, hypertension, hypercholesterolemia, diabetes mellitus, estimated glomerular filtration rate, history of coronary heart disease and history of stroke. Abbreviations: NfL = neurofilament light chain, T-tau = total tau, Aβ = amyloid beta, SD = standard deviation, CI = confidence interval. Associations passing the false discovery rate threshold are denoted with a *. Nominally significant associations (p<0.05) are shaded light gray. | | | | | |

**Supplementary Table 2. Associations between serum immunoglobulins and neuroimaging markers**

|  | Adjusted mean difference (95% CI) | | | |
| --- | --- | --- | --- | --- |
|  | **Total brain volume  (per SD)** | **Gray matter volume  (per SD)** | **White matter volume (per SD)** | **Hippocampal volume (per SD)** |
| Model I |  |  |  |  |
| IgA (per SD) | -0.015 (-0.027 - -0.002) | -0.025 (-0.046 - -0.004) | -0.001 (-0.023 - 0.020) | -0.023 (-0.049 - 0.003) |
| IgG (per SD) | 0.002 (-0.010 - 0.014) | 0.003 (-0.018 - 0.023) | 0.001 (-0.021 - 0.022) | -0.017 (-0.043 - 0.008) |
| IgM (per SD) | -0.003 (-0.015 - 0.010) | -0.003 (-0.023 - 0.017) | -0.002 (-0.023 - 0.019) | 0.007 (-0.018 - 0.031) |
| Model II |  |  |  |  |
| IgA (per SD) | -0.015 (-0.027 - -0.003) | -0.027 (-0.046 - -0.008) | -0.001 (-0.022 - 0.020) | -0.022 (-0.048 - 0.003) |
| IgG (per SD) | -0.003 (-0.015 - 0.009) | -0.005 (-0.025 - 0.014) | -0.001 (-0.022 - 0.021) | -0.020 (-0.046 - 0.007) |
| IgM (per SD) | -0.003 (-0.015 - 0.009) | -0.003 (-0.022 - 0.016) | -0.002 (-0.023 - 0.019) | 0.007 (-0.018 - 0.033) |
| Model III |  |  |  |  |
| IgA (per SD) | -0.013 (-0.025 - -0.002) | -0.026 (-0.045 - -0.007) | 0.002 (-0.019 - 0.023) | -0.023 (-0.049 - 0.003) |
| IgG (per SD) | -0.004 (-0.016 - 0.008) | -0.006 (-0.025 - 0.014) | -0.001 (-0.022 - 0.020) | -0.021 (-0.047 - 0.005) |
| IgM (per SD) | -0.005 (-0.016 - 0.007) | -0.004 (-0.023 - 0.015) | -0.004 (-0.025 - 0.016) | 0.007 (-0.018 - 0.032) |
|  | **White matter hyperintensities (per SD)** | **Fractional anisotropy (per SD)** | **Mean diffusivity  (per SD)** |  |
| Model I |  |  |  |  |
| IgA (per SD) | 0.038 ( 0.010 - 0.066) | -0.025 (-0.061 - 0.011) | 0.021 (-0.006 - 0.047) |  |
| IgG (per SD) | 0.017 (-0.011 - 0.044) | -0.033 (-0.068 - 0.002) | -0.003 (-0.029 - 0.023) |  |
| IgM (per SD) | -0.003 (-0.031 - 0.024) | -0.019 (-0.054 - 0.016) | 0.009 (-0.017 - 0.035) |  |
| Model II |  |  |  |  |
| IgA (per SD) | 0.047 ( 0.016 - 0.077) | -0.032 (-0.067 - 0.003) | 0.031 (-0.001 - 0.063) |  |
| IgG (per SD) | 0.026 (-0.005 - 0.058) | -0.048 (-0.084 - -0.013) | 0.009 (-0.024 - 0.041) |  |
| IgM (per SD) | -0.003 (-0.034 - 0.027) | -0.018 (-0.053 - 0.017) | 0.011 (-0.020 - 0.043) |  |
| Model III |  |  |  |  |
| IgA (per SD) | 0.041 ( 0.012 - 0.071) | -0.028 (-0.062 - 0.007) | 0.027 (-0.004 - 0.059) |  |
| IgG (per SD) | 0.024 (-0.006 - 0.054) | -0.048 (-0.083 - -0.013) | 0.007 (-0.025 - 0.039) |  |
| IgM (per SD) | 0.003 (-0.027 - 0.032) | -0.022 (-0.057 - 0.012) | 0.015 (-0.016 - 0.047) |  |
| Associations are presented as adjusted mean differences (95% confidence interval) for 3139 study participants. Model I is adjusted for age, sex, cohort, intracranial volume and time difference; model II is additionally adjusted for smoking status, alcohol consumption, educational attainment and *APOE*-ε4 carriership; model III is additionally adjusted for BMI, hypertension, hypercholesterolemia, diabetes mellitus, estimated glomerular filtration rate, history of coronary heart disease and history of stroke. Models of white matter hyperintensities, fractional anisotropy and mean diffusivity were additionally adjusted for normal appearing white matter. All neuroimaging markers and serum immunoglobulins were standardized. Abbreviations: SD = standard deviation, CI = confidence interval. Associations passing the false discovery rate threshold are denoted with a *. Nominally significant associations (p<0.05) are shaded light gray. | | | | |

**Supplementary Table 3. Associations between serum immunoglobulins and plasma biomarkers of dementia, full compared to reference range**

| Adjusted mean difference (95% CI) | | | | | | | | | | | |  |
| --- | --- | --- | --- | --- | --- | --- | --- | --- | --- | --- | --- | --- |
|  | **Log2 NfL** | | **Log2 T-tau** | | | **Log2 Aβ-40** | | | **Log2 Aβ-42** | | |  |
|  | **Full range** | **Reference range*** | | **Full range** | **Reference range*** | | **Full range** | **Reference range*** | | **Full range** | **Reference range*** |  |
| Model I |  |  | |  |  | |  |  | |  |  |  |
| IgA (per SD) | 0.008 (-0.011 - 0.028) | 0.015 (-0.006 - 0.037) | | -0.003 (-0.020 - 0.014) | -0.001 (-0.020 - 0.017) | | -0.010 (-0.019 - 0.000) | -0.014 (-0.024 - -0.004) | | -0.014 (-0.028 - 0.000) | -0.009 (-0.023 - 0.006) |  |
| IgG (per SD) | 0.016 (-0.004 - 0.036) | 0.030 ( 0.009 - 0.051) | | -0.001 (-0.018 - 0.016) | -0.010 (-0.029 - 0.008) | | 0.003 (-0.006 - 0.013) | 0.002 (-0.008 - 0.012) | | 0.006 (-0.008 - 0.019) | 0.011 (-0.004 - 0.025) |  |
| IgM (per SD) | -0.007 (-0.028 - 0.013) | 0.004 (-0.017 - 0.025) | | -0.009 (-0.027 - 0.009) | -0.022 (-0.040 - -0.003) | | -0.008 (-0.017 - 0.002) | -0.029 (-0.038 - -0.019) | | -0.010 (-0.024 - 0.004) | -0.038 (-0.052 - -0.023) |  |
| Model II |  |  | |  |  | |  |  | |  |  |  |
| IgA (per SD) | 0.008 (-0.011 - 0.028) | 0.016 (-0.005 - 0.037) | | -0.002 (-0.019 - 0.015) | 0.000 (-0.019 - 0.018) | | -0.009 (-0.019 - 0.000) | -0.015 (-0.025 - -0.004) | | -0.015 (-0.029 - -0.002) | -0.010 (-0.025 - 0.005) |  |
| IgG (per SD) | 0.012 (-0.008 - 0.032) | 0.027 ( 0.006 - 0.049) | | 0.000 (-0.017 - 0.017) | -0.010 (-0.029 - 0.009) | | 0.003 (-0.007 - 0.012) | 0.000 (-0.010 - 0.011) | | 0.002 (-0.012 - 0.016) | 0.007 (-0.008 - 0.021) |  |
| IgM (per SD) | -0.007 (-0.028 - 0.014) | 0.005 (-0.016 - 0.027) | | -0.008 (-0.026 - 0.010) | -0.021 (-0.040 - -0.003) | | -0.007 (-0.017 - 0.003) | -0.028 (-0.038 - -0.018) | | -0.010 (-0.025 - 0.004) | -0.038 (-0.052 - -0.023) |  |
| Model III |  |  | |  |  | |  |  | |  |  |  |
| IgA (per SD) | 0.013 (-0.005 - 0.032) | 0.022 ( 0.002 - 0.043) | | -0.003 (-0.020 - 0.013) | -0.003 (-0.021 - 0.016) | | -0.009 (-0.018 - 0.000) | -0.014 (-0.024 - -0.005) | | -0.012 (-0.025 - 0.001) | -0.008 (-0.022 - 0.007) |  |
| IgG (per SD) | 0.006 (-0.013 - 0.025) | 0.024 ( 0.003 - 0.044) | | -0.005 (-0.022 - 0.012) | -0.013 (-0.031 - 0.006) | | -0.003 (-0.012 - 0.006) | -0.003 (-0.013 - 0.006) | | -0.004 (-0.018 - 0.009) | 0.003 (-0.011 - 0.017) |  |
| IgM (per SD) | -0.011 (-0.031 - 0.008) | -0.002 (-0.022 - 0.018) | | -0.008 (-0.025 - 0.010) | -0.021 (-0.039 - -0.003) | | -0.009 (-0.018 - 0.001) | -0.029 (-0.038 - -0.020) | | -0.012 (-0.026 - 0.002) | -0.039 (-0.053 - -0.025) |  |
| Associations are presented as adjusted mean differences (95% confidence interval) for 3455 study participants in full range and a subset of 2638 participants in reference range. Model I is adjusted for age, sex, study cohort and time difference. Model II is additionally adjusted for smoking status, alcohol consumption, educational attainment and APOE-ε4 carriership. Model III is additionally adjusted for body mass index, hypertension, hypercholesterolemia, diabetes mellitus, estimated glomerular filtration rate, history of coronary heart disease and history of stroke. *Restricted to individuals with reference range of immunoglobulins (0.86-4.76 g/L for IgA, 6.20-15.10 g/L for IgG and 0.28-2.64 g/L for IgM) and no use of medication influencing serum immunoglobulin levels (systemic corticosteroids, antiepileptic drugs, angiotensin converting enzyme inhibitors, cytostatics, immunomodulating or immunosuppressive drugs). Abbreviations: NfL = neurofilament light chain, T-tau = total tau, Aβ = amyloid beta, SD = standard deviation, CI = confidence interval. Nominally significant associations (p<0.05) are shaded light gray. | | | | | | | | | | | | |

**Supplementary Table 4. Associations between serum immunoglobulins and neuroimaging markers, full compared to reference range**

| Adjusted mean difference (95% CI) | | | | | | | | | | | | | | | |
| --- | --- | --- | --- | --- | --- | --- | --- | --- | --- | --- | --- | --- | --- | --- | --- |
|  | **Total brain volume  (per SD)** | | | **Gray matter volume  (per SD)** | | | | **White matter volume  (per SD)** | | | | **Hippocampal volume  (per SD)** | | | |
|  | **Full range** | **Reference range*** | | **Full range** | | **Reference range*** | | **Full range** | | **Reference range*** | | **Full range** | | | **Reference range*** |
| Model I |  |  | |  | |  | |  | |  | |  | | |  |
| IgA (per SD) | -0.015 (-0.027 - -0.002) | -0.010 (-0.023 - 0.002) | | -0.025 (-0.046 - -0.004) | | -0.034 (-0.055 - -0.013) | | -0.001 (-0.023 - 0.020) | | 0.014 (-0.009 - 0.036) | | -0.023 (-0.049 - 0.003) | | | -0.015 (-0.042 - 0.012) |
| IgG (per SD) | 0.002 (-0.010 - 0.014) | 0.009 (-0.004 - 0.022) | | 0.003 (-0.018 - 0.023) | | -0.006 (-0.028 - 0.015) | | 0.001 (-0.021 - 0.022) | | 0.021 (-0.002 - 0.044) | | -0.017 (-0.043 - 0.008) | | | -0.021 (-0.049 - 0.007) |
| IgM (per SD) | -0.003 (-0.015 - 0.010) | 0.001 (-0.013 - 0.014) | | -0.003 (-0.023 - 0.017) | | 0.006 (-0.016 - 0.027) | | -0.002 (-0.023 - 0.019) | | -0.004 (-0.027 - 0.019) | | 0.007 (-0.018 - 0.031) | | | 0.009 (-0.018 - 0.037) |
| Model II |  |  | |  | |  | |  | |  | |  | | |  |
| IgA (per SD) | -0.015 (-0.027 - -0.003) | -0.014 (-0.027 - -0.001) | | -0.027 (-0.046 - -0.008) | | -0.040 (-0.061 - -0.018) | | -0.001 (-0.022 - 0.020) | | 0.014 (-0.010 - 0.038) | | -0.022 (-0.048 - 0.003) | | | -0.016 (-0.046 - 0.013) |
| IgG (per SD) | -0.003 (-0.015 - 0.009) | 0.004 (-0.009 - 0.017) | | -0.005 (-0.025 - 0.014) | | -0.014 (-0.036 - 0.008) | | -0.001 (-0.022 - 0.021) | | 0.020 (-0.004 - 0.044) | | -0.020 (-0.046 - 0.007) | | | -0.025 (-0.055 - 0.004) |
| IgM (per SD) | -0.003 (-0.015 - 0.009) | 0.001 (-0.012 - 0.014) | | -0.003 (-0.022 - 0.016) | | 0.006 (-0.015 - 0.028) | | -0.002 (-0.023 - 0.019) | | -0.005 (-0.029 - 0.019) | | 0.007 (-0.018 - 0.033) | | | 0.010 (-0.019 - 0.039) |
| Model III |  |  | |  | |  | |  | |  | |  | | |  |
| IgA (per SD) | -0.013 (-0.025 - -0.002) | -0.012 (-0.025 - 0.001) | | -0.026 (-0.045 - -0.007) | | -0.040 (-0.062 - -0.019) | | 0.002 (-0.019 - 0.023) | | 0.017 (-0.007 - 0.041) | | -0.023 (-0.049 - 0.003) | | | -0.017 (-0.047 - 0.012) |
| IgG (per SD) | -0.004 (-0.016 - 0.008) | 0.004 (-0.009 - 0.018) | | -0.006 (-0.025 - 0.014) | | -0.014 (-0.036 - 0.008) | | -0.001 (-0.022 - 0.020) | | 0.021 (-0.003 - 0.045) | | -0.021 (-0.047 - 0.005) | | | -0.026 (-0.056 - 0.004) |
| IgM (per SD) | -0.005 (-0.016 - 0.007) | 0.000 (-0.013 - 0.013) | | -0.004 (-0.023 - 0.015) | | 0.006 (-0.015 - 0.028) | | -0.004 (-0.025 - 0.016) | | -0.006 (-0.030 - 0.017) | | 0.007 (-0.018 - 0.032) | | | 0.010 (-0.019 - 0.040) |
|  | **White matter lesions  (per SD)** | | | | **Fractional anisotropy  (per SD)** | | | | **Mean diffusivity  (per SD)** | | | |  |  | |
|  | **Full range** | | **Reference range*** | | **Full range** | | **Reference range*** | | **Full range** | | **Reference range*** | |  |  | |
| Model I |  | |  | |  | |  | |  | |  | |  |  | |
| IgA (per SD) | 0.038 ( 0.010 - 0.066) | | 0.028 (-0.001 - 0.056) | | -0.025 (-0.061 - 0.011) | | -0.013 (-0.051 - 0.025) | | 0.021 (-0.006 - 0.047) | | 0.015 (-0.013 - 0.043) | |  |  | |
| IgG (per SD) | 0.017 (-0.011 - 0.044) | | 0.015 (-0.015 - 0.044) | | -0.033 (-0.068 - 0.002) | | -0.020 (-0.059 - 0.019) | | -0.003 (-0.029 - 0.023) | | 0.000 (-0.029 - 0.028) | |  |  | |
| IgM (per SD) | -0.003 (-0.031 - 0.024) | | 0.001 (-0.028 - 0.030) | | -0.019 (-0.054 - 0.016) | | -0.038 (-0.077 - 0.000) | | 0.009 (-0.017 - 0.035) | | 0.018 (-0.010 - 0.046) | |  |  | |
| Model II |  | |  | |  | |  | |  | |  | |  |  | |
| IgA (per SD) | 0.047 ( 0.016 - 0.077) | | 0.042 ( 0.007 - 0.077) | | -0.032 (-0.067 - 0.003) | | -0.024 (-0.065 - 0.017) | | 0.031 (-0.001 - 0.063) | | 0.030 (-0.008 - 0.069) | |  |  | |
| IgG (per SD) | 0.026 (-0.005 - 0.058) | | 0.029 (-0.006 - 0.065) | | -0.048 (-0.084 - -0.013) | | -0.037 (-0.078 - 0.004) | | 0.009 (-0.024 - 0.041) | | 0.016 (-0.023 - 0.054) | |  |  | |
| IgM (per SD) | -0.003 (-0.034 - 0.027) | | 0.004 (-0.031 - 0.039) | | -0.018 (-0.053 - 0.017) | | -0.042 (-0.082 - -0.002) | | 0.011 (-0.020 - 0.043) | | 0.028 (-0.010 - 0.066) | |  |  | |
| Model III |  | |  | |  | |  | |  | |  | |  |  | |
| IgA (per SD) | 0.041 ( 0.012 - 0.071) | | 0.037 ( 0.002 - 0.071) | | -0.028 (-0.062 - 0.007) | | -0.022 (-0.063 - 0.019) | | 0.027 (-0.004 - 0.059) | | 0.028 (-0.010 - 0.067) | |  |  | |
| IgG (per SD) | 0.024 (-0.006 - 0.054) | | 0.023 (-0.012 - 0.058) | | -0.048 (-0.083 - -0.013) | | -0.035 (-0.076 - 0.006) | | 0.007 (-0.025 - 0.039) | | 0.012 (-0.026 - 0.050) | |  |  | |
| IgM (per SD) | 0.003 (-0.027 - 0.032) | | 0.005 (-0.030 - 0.040) | | -0.022 (-0.057 - 0.012) | | -0.043 (-0.083 - -0.003) | | 0.015 (-0.016 - 0.047) | | 0.030 (-0.008 - 0.068) | |  |  | |
| Associations are presented as adjusted mean differences (95% confidence interval) for 3139 study participants in full range and a subset of 2366 participants in reference range. Model I is adjusted for age, sex, cohort, intracranial volume and time difference; model II is additionally adjusted for smoking status, alcohol consumption, educational attainment and *APOE*-ε4 carriership; model III is additionally adjusted for BMI, hypertension, hypercholesterolemia, diabetes mellitus, estimated glomerular filtration rate, history of coronary heart disease and history of stroke. All neuroimaging markers and serum immunoglobulins were standardized. Models of white matter lesions, fractional anisotropy and mean diffusivity were additionally adjusted for normal appearing white matter. *Restricted to individuals with reference range of immunoglobulins (0.86-4.76 g/L for IgA, 6.20-15.10 g/L for IgG and 0.28-2.64 g/L for IgM) and no use of medication influencing serum immunoglobulin levels (systemic corticosteroids, antiepileptic drugs, angiotensin converting enzyme inhibitors, cytostatics, immunomodulating or immunosuppressive drugs). Abbreviations: SD = standard deviation, CI = confidence interval. Nominally significant associations (p<0.05) are shaded light gray. | | | | | | | | | | | | | | | |

**Supplementary Table 5. Associations between serum immunoglobulins and plasma biomarkers of dementia, stratified by *APOE*-ε4 carriership**

| Adjusted mean difference (95% CI) | | | | | | | | | | | |  |
| --- | --- | --- | --- | --- | --- | --- | --- | --- | --- | --- | --- | --- |
|  | **Log2 NfL** | | **Log2 T-tau** | | | **Log2 Aβ-40** | | | **Log2 Aβ-42** | | |  |
|  | **Non-carrier** | **Carrier** | | **Non-carrier** | **Carrier** | | **Non-carrier** | **Carrier** | | **Non-carrier** | **Carrier** |  |
| Model I |  |  | |  |  | |  |  | |  |  |  |
| IgA (per SD) | 0.002 (-0.022 - 0.026) | 0.022 (-0.016 - 0.059) | | -0.016 (-0.037 - 0.004) | 0.030 (-0.002 - 0.061) | | -0.013 (-0.024 - -0.001) | 0.000 (-0.017 - 0.016) | | -0.023 (-0.040 - -0.007) | 0.013 (-0.013 - 0.038) |  |
| IgG (per SD) | 0.022 (-0.002 - 0.045) | 0.002 (-0.037 - 0.041) | | -0.003 (-0.023 - 0.017) | 0.008 (-0.024 - 0.041) | | 0.002 (-0.010 - 0.013) | 0.012 (-0.005 - 0.030) | | 0.003 (-0.013 - 0.019) | 0.016 (-0.011 - 0.042) |  |
| IgM (per SD) | -0.012 (-0.036 - 0.013) | 0.001 (-0.042 - 0.044) | | -0.013 (-0.035 - 0.008) | 0.006 (-0.030 - 0.042) | | -0.013 (-0.025 - -0.001) | 0.008 (-0.011 - 0.028) | | -0.011 (-0.028 - 0.006) | -0.012 (-0.043 - 0.019) |  |
| Model II |  |  | |  |  | |  |  | |  |  |  |
| IgA (per SD) | 0.002 (-0.021 - 0.026) | 0.022 (-0.016 - 0.060) | | -0.016 (-0.037 - 0.004) | 0.034 ( 0.003 - 0.066) | | -0.013 (-0.025 - -0.001) | 0.001 (-0.016 - 0.018) | | -0.024 (-0.041 - -0.008) | 0.012 (-0.014 - 0.038) |  |
| IgG (per SD) | 0.017 (-0.006 - 0.041) | -0.002 (-0.042 - 0.038) | | -0.003 (-0.024 - 0.017) | 0.010 (-0.023 - 0.044) | | 0.000 (-0.011 - 0.012) | 0.015 (-0.003 - 0.033) | | 0.000 (-0.017 - 0.016) | 0.013 (-0.014 - 0.041) |  |
| IgM (per SD) | -0.010 (-0.035 - 0.014) | -0.002 (-0.045 - 0.042) | | -0.012 (-0.033 - 0.009) | 0.004 (-0.032 - 0.040) | | -0.013 (-0.025 - -0.001) | 0.009 (-0.011 - 0.028) | | -0.010 (-0.027 - 0.006) | -0.012 (-0.043 - 0.019) |  |
| Model III |  |  | |  |  | |  |  | |  |  |  |
| IgA (per SD) | 0.008 (-0.014 - 0.031) | 0.021 (-0.015 - 0.057) | | -0.015 (-0.036 - 0.005) | 0.030 (-0.002 - 0.061) | | -0.010 (-0.021 - 0.001) | -0.005 (-0.021 - 0.011) | | -0.018 (-0.034 - -0.002) | 0.006 (-0.019 - 0.031) |  |
| IgG (per SD) | 0.009 (-0.013 - 0.031) | -0.002 (-0.041 - 0.037) | | -0.009 (-0.029 - 0.011) | 0.012 (-0.022 - 0.045) | | -0.006 (-0.017 - 0.005) | 0.008 (-0.009 - 0.025) | | -0.007 (-0.023 - 0.008) | 0.006 (-0.021 - 0.032) |  |
| IgM (per SD) | -0.016 (-0.039 - 0.007) | -0.005 (-0.046 - 0.036) | | -0.012 (-0.033 - 0.008) | 0.005 (-0.030 - 0.041) | | -0.014 (-0.025 - -0.003) | 0.005 (-0.013 - 0.024) | | -0.012 (-0.028 - 0.004) | -0.014 (-0.044 - 0.015) |  |
| Associations are presented as adjusted mean differences (95% confidence interval) for 2432 non-carriers and 920 carriers of the *APOE*-ε4 allele. Model I is adjusted for age, sex, study cohort and time difference. Model II is additionally adjusted for smoking status, alcohol consumption and educational attainment. Model III is additionally adjusted for body mass index, hypertension, hypercholesterolemia, diabetes mellitus, estimated glomerular filtration rate, history of coronary heart disease and history of stroke. Abbreviations: NfL = neurofilament light chain, T-tau = total tau, Aβ = amyloid beta, SD = standard deviation, CI = confidence interval. Nominally significant associations (p<0.05) are shaded light gray. | | | | | | | | | | | | |
|  | | | | | | | | | | | | |

**Supplementary Table 6. Associations between serum immunoglobulins and neuroimaging markers, stratified by *APOE*-ε4 carriership**

| Adjusted mean difference (95% CI) | | | | | | | | | | | | | | | |
| --- | --- | --- | --- | --- | --- | --- | --- | --- | --- | --- | --- | --- | --- | --- | --- |
|  | **Total brain volume  (per SD)** | | | **Gray matter volume  (per SD)** | | | | **White matter volume  (per SD)** | | | | **Hippocampal volume  (per SD)** | | | |
|  | **Non-carrier** | **Carrier** | | **Non-carrier** | | **Carrier** | | **Non-carrier** | | **Carrier** | | **Non-carrier** | | | **Carrier** |
| Model I |  |  | |  | |  | |  | |  | |  | | |  |
| IgA (per SD) | -0.017 (-0.040 - 0.005) | -0.013 (-0.029 - 0.002) | | -0.015 (-0.052 - 0.022) | | -0.028 (-0.053 - -0.002) | | -0.015 (-0.054 - 0.024) | | 0.002 (-0.025 - 0.030) | | 0.000 (-0.047 - 0.048) | | | -0.023 (-0.054 - 0.009) |
| IgG (per SD) | 0.001 (-0.020 - 0.023) | 0.006 (-0.010 - 0.022) | | -0.001 (-0.036 - 0.034) | | 0.003 (-0.023 - 0.029) | | 0.003 (-0.034 - 0.040) | | 0.008 (-0.020 - 0.035) | | 0.006 (-0.039 - 0.051) | | | -0.012 (-0.044 - 0.020) |
| IgM (per SD) | -0.009 (-0.028 - 0.010) | 0.004 (-0.012 - 0.021) | | 0.002 (-0.030 - 0.034) | | -0.004 (-0.031 - 0.023) | | -0.017 (-0.051 - 0.016) | | 0.011 (-0.018 - 0.040) | | 0.001 (-0.040 - 0.041) | | | 0.015 (-0.018 - 0.048) |
| Model II |  |  | |  | |  | |  | |  | |  | | |  |
| IgA (per SD) | -0.020 (-0.042 - 0.003) | -0.013 (-0.028 - 0.001) | | -0.018 (-0.055 - 0.019) | | -0.029 (-0.053 - -0.006) | | -0.016 (-0.057 - 0.024) | | 0.004 (-0.021 - 0.030) | | -0.001 (-0.052 - 0.051) | | | -0.022 (-0.053 - 0.009) |
| IgG (per SD) | -0.004 (-0.028 - 0.019) | 0.001 (-0.014 - 0.016) | | -0.009 (-0.047 - 0.029) | | -0.006 (-0.030 - 0.018) | | 0.001 (-0.040 - 0.043) | | 0.007 (-0.019 - 0.033) | | 0.006 (-0.047 - 0.058) | | | -0.015 (-0.047 - 0.017) |
| IgM (per SD) | -0.011 (-0.034 - 0.011) | 0.003 (-0.011 - 0.018) | | 0.000 (-0.037 - 0.036) | | -0.004 (-0.027 - 0.019) | | -0.019 (-0.059 - 0.021) | | 0.009 (-0.016 - 0.034) | | 0.001 (-0.049 - 0.051) | | | 0.013 (-0.017 - 0.044) |
| Model III |  |  | |  | |  | |  | |  | |  | | |  |
| IgA (per SD) | -0.015 (-0.038 - 0.007) | -0.012 (-0.026 - 0.003) | | -0.016 (-0.054 - 0.021) | | -0.032 (-0.055 - -0.008) | | -0.010 (-0.051 - 0.030) | | 0.009 (-0.017 - 0.035) | | 0.003 (-0.049 - 0.054) | | | -0.022 (-0.053 - 0.009) |
| IgG (per SD) | -0.003 (-0.026 - 0.019) | 0.001 (-0.014 - 0.015) | | -0.008 (-0.046 - 0.030) | | -0.008 (-0.032 - 0.016) | | 0.002 (-0.039 - 0.043) | | 0.008 (-0.017 - 0.034) | | -0.002 (-0.055 - 0.050) | | | -0.014 (-0.046 - 0.018) |
| IgM (per SD) | -0.014 (-0.036 - 0.008) | 0.002 (-0.012 - 0.017) | | -0.002 (-0.038 - 0.035) | | -0.004 (-0.027 - 0.019) | | -0.023 (-0.063 - 0.017) | | 0.008 (-0.017 - 0.033) | | -0.004 (-0.054 - 0.046) | | | 0.015 (-0.015 - 0.045) |
|  | **White matter lesions  (per SD)** | | | | **Fractional anisotropy  (per SD)** | | | | **Mean diffusivity  (per SD)** | | | |  |  | |
|  | **Non-carrier** | | **Carrier** | | **Non-carrier** | | **Carrier** | | **Non-carrier** | | **Carrier** | |  |  | |
| Model I |  | |  | |  | |  | |  | |  | |  |  | |
| IgA (per SD) | 0.023 (-0.027 - 0.073) | | 0.048 ( 0.013 - 0.083) | | -0.032 (-0.098 - 0.034) | | -0.009 (-0.054 - 0.035) | | 0.034 (-0.015 - 0.084) | | 0.009 (-0.024 - 0.041) | |  |  | |
| IgG (per SD) | -0.012 (-0.060 - 0.035) | | 0.037 ( 0.001 - 0.072) | | -0.056 (-0.118 - 0.006) | | -0.014 (-0.059 - 0.031) | | 0.000 (-0.047 - 0.046) | | -0.012 (-0.045 - 0.021) | |  |  | |
| IgM (per SD) | 0.004 (-0.039 - 0.047) | | -0.009 (-0.046 - 0.028) | | -0.006 (-0.064 - 0.051) | | -0.022 (-0.069 - 0.025) | | 0.020 (-0.023 - 0.064) | | 0.004 (-0.030 - 0.038) | |  |  | |
| Model II |  | |  | |  | |  | |  | |  | |  |  | |
| IgA (per SD) | 0.031 (-0.028 - 0.090) | | 0.056 ( 0.019 - 0.094) | | -0.043 (-0.111 - 0.024) | | -0.016 (-0.059 - 0.027) | | 0.051 (-0.013 - 0.114) | | 0.014 (-0.024 - 0.053) | |  |  | |
| IgG (per SD) | -0.013 (-0.074 - 0.047) | | 0.049 ( 0.010 - 0.087) | | -0.081 (-0.149 - -0.013) | | -0.026 (-0.070 - 0.017) | | 0.011 (-0.053 - 0.075) | | -0.003 (-0.042 - 0.036) | |  |  | |
| IgM (per SD) | 0.004 (-0.055 - 0.062) | | -0.007 (-0.044 - 0.030) | | -0.006 (-0.074 - 0.062) | | -0.018 (-0.060 - 0.024) | | 0.030 (-0.034 - 0.094) | | 0.004 (-0.034 - 0.042) | |  |  | |
| Model III |  | |  | |  | |  | |  | |  | |  |  | |
| IgA (per SD) | 0.027 (-0.030 - 0.084) | | 0.049 ( 0.011 - 0.086) | | -0.036 (-0.103 - 0.031) | | -0.010 (-0.053 - 0.033) | | 0.044 (-0.019 - 0.107) | | 0.010 (-0.028 - 0.049) | |  |  | |
| IgG (per SD) | -0.011 (-0.069 - 0.047) | | 0.042 ( 0.005 - 0.080) | | -0.080 (-0.148 - -0.012) | | -0.023 (-0.067 - 0.021) | | 0.008 (-0.056 - 0.072) | | -0.007 (-0.046 - 0.032) | |  |  | |
| IgM (per SD) | 0.020 (-0.036 - 0.076) | | -0.005 (-0.042 - 0.031) | | -0.015 (-0.083 - 0.053) | | -0.020 (-0.062 - 0.022) | | 0.038 (-0.026 - 0.102) | | 0.007 (-0.031 - 0.044) | |  |  | |
| Associations are presented as adjusted mean differences (95% confidence interval) for 2065 non-carriers and 875 carriers of the *APOE*-ε4 allele. Model I is adjusted for age, sex, cohort, intracranial volume and time difference; model II is additionally adjusted for smoking status, alcohol consumption and educational attainment; model III is additionally adjusted for BMI, hypertension, hypercholesterolemia, diabetes mellitus, estimated glomerular filtration rate, history of coronary heart disease and history of stroke. All neuroimaging markers and serum immunoglobulins were standardized. Models of white matter lesions, fractional anisotropy and mean diffusivity were additionally adjusted for normal appearing white matter. Abbreviations: SD = standard deviation, CI = confidence interval. Nominally significant associations (p<0.05) are shaded light gray. | | | | | | | | | | | | | | | |

**Supplementary Table 7. Associations between serum immunoglobulins and plasma biomarkers of dementia, stratified by sex**

| Adjusted mean difference (95% CI) | | | | | | | | | | | |  |
| --- | --- | --- | --- | --- | --- | --- | --- | --- | --- | --- | --- | --- |
|  | **Log2 NfL** | | **Log2 T-tau** | | | **Log2 Aβ-40** | | | **Log2 Aβ-42** | | |  |
|  | **Men** | **Women** | | **Men** | **Women** | | **Men** | **Women** | | **Men** | **Women** |  |
| Model I |  |  | |  |  | |  |  | |  |  |  |
| IgA (per SD) | 0.011 (-0.020 - 0.041) | 0.006 (-0.019 - 0.032) | | 0.007 (-0.020 - 0.033) | -0.012 (-0.034 - 0.009) | | 0.001 (-0.014 - 0.016) | -0.019 (-0.031 - -0.006) | | 0.004 (-0.016 - 0.024) | -0.030 (-0.049 - -0.011) |  |
| IgG (per SD) | 0.018 (-0.013 - 0.049) | 0.014 (-0.011 - 0.039) | | 0.017 (-0.010 - 0.044) | -0.014 (-0.035 - 0.008) | | 0.002 (-0.013 - 0.017) | 0.004 (-0.008 - 0.016) | | 0.005 (-0.015 - 0.025) | 0.006 (-0.012 - 0.024) |  |
| IgM (per SD) | 0.016 (-0.020 - 0.051) | -0.024 (-0.047 - -0.001) | | -0.023 (-0.055 - 0.008) | 0.003 (-0.017 - 0.022) | | 0.004 (-0.014 - 0.021) | -0.016 (-0.027 - -0.005) | | 0.005 (-0.019 - 0.029) | -0.020 (-0.037 - -0.004) |  |
| Model II |  |  | |  |  | |  |  | |  |  |  |
| IgA (per SD) | 0.010 (-0.020 - 0.041) | 0.007 (-0.019 - 0.032) | | 0.008 (-0.019 - 0.035) | -0.011 (-0.032 - 0.011) | | 0.002 (-0.013 - 0.016) | -0.019 (-0.031 - -0.007) | | 0.001 (-0.019 - 0.021) | -0.029 (-0.048 - -0.010) |  |
| IgG (per SD) | 0.011 (-0.020 - 0.042) | 0.011 (-0.015 - 0.037) | | 0.020 (-0.008 - 0.047) | -0.014 (-0.035 - 0.008) | | 0.002 (-0.013 - 0.017) | 0.002 (-0.010 - 0.015) | | 0.000 (-0.020 - 0.021) | 0.002 (-0.017 - 0.021) |  |
| IgM (per SD) | 0.014 (-0.021 - 0.050) | -0.024 (-0.047 - -0.001) | | -0.023 (-0.054 - 0.009) | 0.004 (-0.016 - 0.023) | | 0.003 (-0.014 - 0.021) | -0.016 (-0.027 - -0.005) | | 0.003 (-0.021 - 0.027) | -0.021 (-0.037 - -0.004) |  |
| Model III |  |  | |  |  | |  |  | |  |  |  |
| IgA (per SD) | 0.015 (-0.014 - 0.043) | 0.011 (-0.014 - 0.035) | | 0.008 (-0.018 - 0.035) | -0.014 (-0.036 - 0.007) | | 0.003 (-0.011 - 0.017) | -0.020 (-0.032 - -0.008) | | 0.005 (-0.013 - 0.024) | -0.028 (-0.046 - -0.009) |  |
| IgG (per SD) | 0.005 (-0.025 - 0.035) | 0.004 (-0.021 - 0.028) | | 0.015 (-0.012 - 0.043) | -0.020 (-0.042 - 0.001) | | -0.004 (-0.018 - 0.010) | -0.004 (-0.016 - 0.007) | | -0.007 (-0.026 - 0.012) | -0.004 (-0.022 - 0.014) |  |
| IgM (per SD) | 0.006 (-0.027 - 0.040) | -0.026 (-0.048 - -0.004) | | -0.026 (-0.057 - 0.005) | 0.004 (-0.015 - 0.024) | | 0.000 (-0.016 - 0.016) | -0.016 (-0.027 - -0.006) | | 0.000 (-0.022 - 0.022) | -0.021 (-0.038 - -0.005) |  |
| Associations are presented as adjusted mean differences (95% confidence interval) for 1976 women and 1479 men. Model I is adjusted for age, study cohort and time difference. Model II is additionally adjusted for smoking status, alcohol consumption, educational attainment and APOE-ε4 carriership. Model III is additionally adjusted for body mass index, hypertension, hypercholesterolemia, diabetes mellitus, estimated glomerular filtration rate, history of coronary heart disease and history of stroke. Abbreviations: NfL = neurofilament light chain, T-tau = total tau, Aβ = amyloid beta, SD = standard deviation, CI = confidence interval. Nominally significant associations (p<0.05) are shaded light gray. | | | | | | | | | | | | |
|  | | | | | | | | | | | | |

**Supplementary Table 8. Associations between serum immunoglobulins and neuroimaging markers, stratified by sex**

| Adjusted mean difference (95% CI) | | | | | | | | | | | | | | | |
| --- | --- | --- | --- | --- | --- | --- | --- | --- | --- | --- | --- | --- | --- | --- | --- |
|  | **Total brain volume  (per SD)** | | | **Gray matter volume  (per SD)** | | | | **White matter volume  (per SD)** | | | | **Hippocampal volume  (per SD)** | | | |
|  | **Men** | **Women** | | **Men** | | **Women** | | **Men** | | **Women** | | **Men** | | | **Women** |
| Model I |  |  | |  | |  | |  | |  | |  | | |  |
| IgA (per SD) | -0.023 (-0.044 - -0.002) | -0.007 (-0.027 - 0.012) | | -0.021 (-0.056 - 0.014) | | -0.031 (-0.062 - 0.000) | | -0.017 (-0.053 - 0.019) | | 0.015 (-0.016 - 0.046) | | -0.041 (-0.084 - 0.001) | | | -0.010 (-0.047 - 0.026) |
| IgG (per SD) | 0.001 (-0.019 - 0.022) | 0.005 (-0.014 - 0.024) | | 0.008 (-0.026 - 0.042) | | 0.000 (-0.030 - 0.031) | | -0.005 (-0.040 - 0.030) | | 0.008 (-0.023 - 0.038) | | -0.023 (-0.064 - 0.019) | | | -0.016 (-0.053 - 0.020) |
| IgM (per SD) | 0.002 (-0.020 - 0.024) | -0.004 (-0.022 - 0.014) | | 0.003 (-0.032 - 0.039) | | -0.004 (-0.032 - 0.024) | | 0.000 (-0.037 - 0.037) | | -0.003 (-0.031 - 0.025) | | 0.026 (-0.017 - 0.069) | | | -0.013 (-0.046 - 0.021) |
| Model II |  |  | |  | |  | |  | |  | |  | | |  |
| IgA (per SD) | -0.021 (-0.040 - -0.001) | -0.010 (-0.029 - 0.008) | | -0.020 (-0.053 - 0.012) | | -0.036 (-0.065 - -0.007) | | -0.015 (-0.049 - 0.019) | | 0.015 (-0.015 - 0.045) | | -0.042 (-0.084 - 0.000) | | | -0.008 (-0.046 - 0.030) |
| IgG (per SD) | 0.000 (-0.020 - 0.020) | -0.006 (-0.026 - 0.013) | | 0.006 (-0.027 - 0.038) | | -0.016 (-0.046 - 0.014) | | -0.005 (-0.039 - 0.029) | | 0.004 (-0.027 - 0.035) | | -0.025 (-0.067 - 0.017) | | | -0.019 (-0.059 - 0.020) |
| IgM (per SD) | 0.002 (-0.018 - 0.021) | -0.005 (-0.023 - 0.014) | | 0.004 (-0.028 - 0.036) | | -0.004 (-0.033 - 0.025) | | -0.001 (-0.034 - 0.033) | | -0.004 (-0.034 - 0.026) | | 0.024 (-0.016 - 0.065) | | | -0.014 (-0.051 - 0.024) |
| Model III |  |  | |  | |  | |  | |  | |  | | |  |
| IgA (per SD) | -0.019 (-0.039 - 0.000) | -0.007 (-0.025 - 0.012) | | -0.021 (-0.053 - 0.012) | | -0.033 (-0.063 - -0.004) | | -0.012 (-0.046 - 0.022) | | 0.019 (-0.011 - 0.049) | | -0.044 (-0.086 - -0.002) | | | -0.008 (-0.046 - 0.030) |
| IgG (per SD) | -0.001 (-0.021 - 0.018) | -0.006 (-0.025 - 0.013) | | 0.005 (-0.028 - 0.037) | | -0.015 (-0.046 - 0.015) | | -0.007 (-0.041 - 0.027) | | 0.004 (-0.027 - 0.036) | | -0.027 (-0.069 - 0.015) | | | -0.020 (-0.060 - 0.020) |
| IgM (per SD) | -0.002 (-0.021 - 0.017) | -0.006 (-0.024 - 0.012) | | 0.003 (-0.029 - 0.035) | | -0.005 (-0.034 - 0.024) | | -0.006 (-0.040 - 0.027) | | -0.005 (-0.034 - 0.025) | | 0.022 (-0.018 - 0.063) | | | -0.014 (-0.051 - 0.024) |
|  | **White matter lesions  (per SD)** | | | | **Fractional anisotropy  (per SD)** | | | | **Mean diffusivity  (per SD)** | | | |  |  | |
|  | **Men** | | **Women** | | **Men** | | **Women** | | **Men** | | **Women** | |  |  | |
| Model I |  | |  | |  | |  | |  | |  | |  |  | |
| IgA (per SD) | 0.028 (-0.014 - 0.070) | | 0.047 ( 0.009 - 0.084) | | -0.007 (-0.062 - 0.048) | | -0.040 (-0.089 - 0.008) | | 0.026 (-0.015 - 0.067) | | 0.021 (-0.014 - 0.056) | |  |  | |
| IgG (per SD) | 0.016 (-0.026 - 0.057) | | 0.017 (-0.020 - 0.054) | | -0.025 (-0.079 - 0.029) | | -0.036 (-0.084 - 0.011) | | -0.002 (-0.042 - 0.039) | | -0.003 (-0.037 - 0.032) | |  |  | |
| IgM (per SD) | -0.036 (-0.080 - 0.007) | | 0.030 (-0.004 - 0.064) | | 0.025 (-0.031 - 0.081) | | -0.067 (-0.111 - -0.022) | | -0.012 (-0.054 - 0.030) | | 0.033 ( 0.001 - 0.065) | |  |  | |
| Model II |  | |  | |  | |  | |  | |  | |  |  | |
| IgA (per SD) | 0.038 (-0.007 - 0.083) | | 0.051 ( 0.010 - 0.093) | | -0.012 (-0.065 - 0.040) | | -0.048 (-0.096 - -0.001) | | 0.033 (-0.016 - 0.081) | | 0.032 (-0.010 - 0.074) | |  |  | |
| IgG (per SD) | 0.025 (-0.021 - 0.070) | | 0.024 (-0.019 - 0.068) | | -0.033 (-0.086 - 0.020) | | -0.060 (-0.109 - -0.011) | | 0.005 (-0.043 - 0.054) | | 0.013 (-0.031 - 0.057) | |  |  | |
| IgM (per SD) | -0.037 (-0.082 - 0.007) | | 0.038 (-0.003 - 0.079) | | 0.026 (-0.026 - 0.078) | | -0.072 (-0.120 - -0.024) | | -0.014 (-0.062 - 0.033) | | 0.044 ( 0.001 - 0.086) | |  |  | |
| Model III |  | |  | |  | |  | |  | |  | |  |  | |
| IgA (per SD) | 0.037 (-0.006 - 0.081) | | 0.041 ( 0.000 - 0.082) | | -0.013 (-0.065 - 0.039) | | -0.041 (-0.088 - 0.007) | | 0.034 (-0.014 - 0.082) | | 0.024 (-0.018 - 0.066) | |  |  | |
| IgG (per SD) | 0.023 (-0.020 - 0.067) | | 0.021 (-0.021 - 0.064) | | -0.036 (-0.088 - 0.016) | | -0.056 (-0.105 - -0.007) | | 0.006 (-0.042 - 0.054) | | 0.008 (-0.036 - 0.051) | |  |  | |
| IgM (per SD) | -0.027 (-0.070 - 0.016) | | 0.040 (-0.001 - 0.080) | | 0.020 (-0.031 - 0.071) | | -0.074 (-0.122 - -0.027) | | -0.008 (-0.055 - 0.039) | | 0.045 ( 0.003 - 0.088) | |  |  | |
| Associations are presented as adjusted mean differences (95% confidence interval) for 1709 women and 1430 men. Model I is adjusted for age, cohort, intracranial volume and time difference; model II is additionally adjusted for smoking status, alcohol consumption educational attainment and *APOE*-ε4 carriership; model III is additionally adjusted for BMI, hypertension, hypercholesterolemia, diabetes mellitus, estimated glomerular filtration rate, history of coronary heart disease and history of stroke. All neuroimaging markers and serum immunoglobulins were standardized. Models of white matter lesions, fractional anisotropy and mean diffusivity were additionally adjusted for normal appearing white matter. Abbreviations: SD = standard deviation, CI = confidence interval. Nominally significant associations (p<0.05) are shaded light gray. | | | | | | | | | | | | | | | |
